# Multivariate analysis of yield, physiological, and biochemical traits in peanut (*Arachis hypogaea* L.) cultivars

**DOI:** 10.3389/fpls.2026.1850930

**Published:** 2026-06-08

**Authors:** Tahsin Beycioglu

**Affiliations:** Field Crops Department, Faculty of Agriculture, Pamukkale University, Denizli, Türkiye

**Keywords:** antioxidant capacity, fatty acid composition, leaf area index, Pod yield, Total Chlorophyll

## Abstract

**Introduction:**

This study was carried out for two years to determine the performance of different peanut (*Arachis hypogaea* L.) cultivars in terms of yield, quality, physiological parameters, and bioactive compounds, and to identify the relationships among these traits using multivariate analysis methods.

**Methods:**

In the study, the pod yield, oil and protein content, fatty acid composition, leaf area index (LAI), chlorophyll content, total phenolic content, flavonoid content, and antioxidant activity parameters of 10 different peanut cultivars were examined.

**Results:**

Analysis of variance results indicated that for all parameters examined, the effects of cultivar, year, and the C × Y interaction were statistically significant (*p* < 0.01). The highest pod yield was obtained from the Osmaniye 2005 (4815.70 kg ha⁻¹) and Sultan (4683.50 kg ha⁻¹) cultivars, while the highest oil content (54.95%) and oleic acid percentage (68.44%) were obtained from the Brantley cultivar. The negative correlation observed between oleic and linoleic acid ratios indicated that cultivars with high oleic acid content exhibited higher oxidative stability. Physiological analyses revealed that chlorophyll content during the pod development stage had a more pronounced positive effect on yield components compared to the flowering stage. Principal Component Analysis (PCA) and Heat Map results showed that the cultivars were grouped into two main clusters focused on yield and quality. Osmaniye 2005 and Sultan cultivars were identified as the most superior parental candidates for breeding programs in terms of yield, while Brantley was identified as the most superior candidate for high oil quality. Additionally, 3D surface response modeling revealed a strong correlation between yield increase and high photosynthetic capacity and antioxidant activity.

**Discussion:**

Consequently, this study may provide critical selection criteria and genetic materials for the development of new peanut cultivars with both high yield and superior oil quality.

## 1 Introduction

Peanut (*Arachis hypogaea* L.) is an important oilseed crop belonging to the Leguminosae family and is cultivated over vast areas worldwide. This self-pollinating plant species has a chromosome number of 2n = 40 (Harika et al., 2025). As a global crop, its seeds are rich in easily digestible protein (22–30%) and high-quality edible oil (44–56%), as well as essential minerals and vitamins. It is distinguished by its high concentration of linoleic and oleic acids (Baraker et al., 2017; Yol et al., 2017). Its seeds are rich in monounsaturated fatty acids (50%) and support cardiovascular health while lowering cholesterol levels (Çiftçi and Suna, 2022). Peanuts possess a rich biochemical profile ranging from peptides to flavonoids and phenolic compounds. Peanuts hold a significant place in Turkey and worldwide. In 2024, global peanut seed production reached 57 million tons, with Turkey ranking 27th globally with a production of 247,000 tons. China ranked first with 20 million tons, followed by India (12 million tons), Nigeria (4.5 million tons), and the United States (3 million tons) (FAOSTAT, 2026). According to data from the 2024 growing season, peanut production in Turkey was carried out on an area of 57,641.80 hectar. The yield per unit area was recorded as 4280 kg ha^−1^. However, interregional yield differences highlight the importance of cultivar-environment interaction. According to 2024 crop production values, production in the Aegean region amounted to 2,618 tons. The fact that the region’s yield value (3890 kg ha^−1^) is 9.2% below the Turkish average indicates the need to select cultivars suitable for the region (TUIK, 2026). Peanut yield is influenced by the genetic potential of the selected cultivar and the agricultural management practices applied. The selection of cultivars that are adaptable and resistant to diseases and pests is among the factors that increase yield (Sagvekar et al., 2017; Kishlyan et al., 2020). Chlorophyll is the primary substance used by green plants to absorb, convert, and transmit light energy through photosynthesis, and it is associated with processes related to plant growth and aging, photosynthetic capacity, disease, nutrition, and environmental stress (Viña et al., 2011). Leaf area measurement is a crucial parameter that provides important information about plant health, development, and productivity. Leaves play a key role in the release of water vapor into the atmosphere through photosynthesis and in general growth processes (Gokkus and Gokkus, 2024). Therefore, leaf area measurement is a technique widely used in plant physiology, ecology, and agricultural research. Plant yield is related to the photosynthesis rate corresponding to each leaf and its associated leaves (Haghshenas and Emam, 2022). The effect of leaf area on plant biomass is an important indicator of plant development and yield. Consequently, in most agricultural studies, leaf area has been correlated with other parameters (Haghshenas and Emam, 2022; Waldner et al., 2019). Biochemical contents in peanuts can vary significantly depending on genotype, environmental conditions, and cultivation practices (Devi et al., 2025; Gokkus, 2025). In peanut cultivation, achieving high yields and profitable production per unit area requires not only timely and technically appropriate cultural practices but also the identification of cultivars suitable for the specific location and growing conditions. For this reason, adaptation studies are being conducted in different locations to identify cultivars and lines suitable for local environmental conditions (Söğüt et al., 2002; Çalışkan and Arıoğlu, 2004; Arslan et al., 2005; Kurt et al., 2009). Global climate change and the increasing need for food security necessitate the development of high-yielding and stress-tolerant cultivars and the determination of their regional adaptations (Pandey et al., 2022; Puppala et al., 2023).

There are no studies evaluating the multi-year performance of peanut cultivars and cultivar × year interactions using multivariate statistical analyses under the ecological conditions of Denizli. The aim of this study is to evaluate the yield, physiological, and biochemical characteristics of different peanut cultivars under the ecological conditions of Denizli-Çal using two years of data. Additionally, the study aims to determine the cultivar × year interactions and identify the relationships among the cultivars. The findings are expected to be used to select the cultivars that demonstrate the highest adaptation to the regional ecology and offer sustainable productivity.

## 2 Materials and methods

### 2.1 Site description

**Figure 1:**
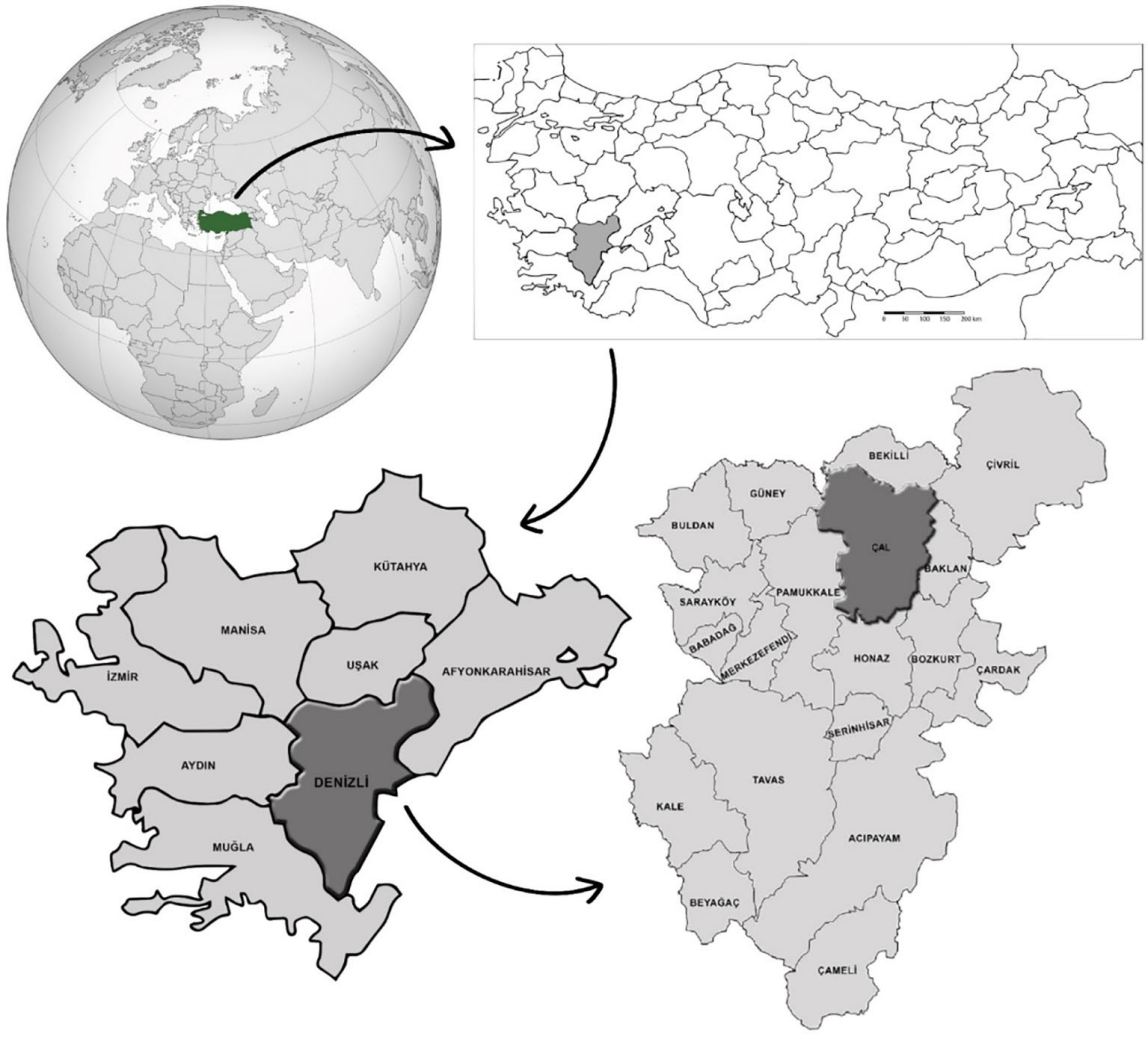
Geographical location of the trial field where the studied peanut cultivars were grown.

The research was conducted at the Research and Application Center of the Faculty of Agriculture at Pamukkale University, located in the Çal district of Denizli Province in the Aegean Region. The experimental area was located at coordinates 38°05’57”north altitude and 29°25’04” east longitude (**Figure 1**).

Soil analysis results for the research area at a depth of 0–30 cm indicate low salinity and a slightly alkaline reaction. Soils from the trial area have high lime content and low organic matter content. Potassium and phosphorus levels are sufficient in terms of nutrient content. However, due to high pH and lime content, low concentrations of microelements such as iron, copper, manganese, and zinc have been detected (**Table 1**).

**Table 1:** Results of soil analysis (0–30 cm depth) of the research area.

Location	EC us (µS cm^-1^)	pH	Lime (%)	K (mg kg^-1^)	P (mg kg^-1^)	O.M (%)	Cu (mg kg^-1^)	Fe (mg kg^-1^)	Zn (mg kg^-1^)	Mn (mg kg^-1^)
Denizli-Çal	450.00	8.30	25.77	365.00	26.37	1.17	1.70	5.40	0.40	11.40

**Figure 2:**
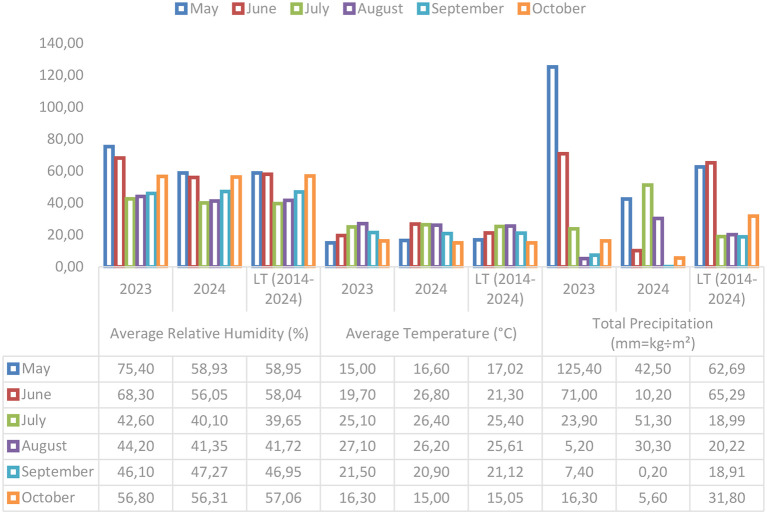
Climate parameters of the research field (2023, 2024 and long-year average).

The climate data for the 2023 and 2024 growing seasons in the experimental area, when compared to the long-term averages (2014–2024), show that both production years were affected by extreme weather events. In particular, the rainfall amount in May 2023 (125.4 mm) was approximately twice that of both 2024 (42.5 mm) and the long-term average (62.69 mm). In terms of temperature data, June 2024 (26.8 °C) was significantly warmer than both the previous year (19.7 °C) and the long-term average (21.3 °C). In terms of precipitation patterns, the 51.3 mm of rainfall recorded in July 2024 was well above the long-term average (18.99 mm); however, the drop in precipitation to 5.6 mm in September is noteworthy. Relative humidity values were higher in May (75.4%) and June (68.3%) of 2023 but showed a decline in 2024. In summary, the 2024 growing season was characterized by a higher total temperature and an irregular precipitation regime compared to 2023 (**Figure 2**).

### 2.2 Experimental materials and design

The study was carried out for two years during the 2023 and 2024 main growing seasons, using a randomized block design with three replications. The study utilized the following cultivars: Arıoğlu 2003, Batem 5025, Batem Cihangir, Brantley, Flower-22, Halisbey, NC-7, Osmaniye 2005, Sultan, and Wilson. Peanut seeds were planted at a depth of 5–6 cm, with approximately 9,500 plants per decare. Plantings were arranged in 4 rows with a row length of 5 m (5.0 m × 2.8 m = 14 m^2^), a row spacing of 70 cm, and a plant spacing of 15 cm within the row. Prior to planting, 300 kg ha^-1^ of 18-46–0 DAP fertilizer was applied, and approximately 60 days after emergence, 150 kg ha^-1^ of urea fertilizer (46% N) was applied using a fertilizer spreader. Cultural practices were carried out in accordance with the phenological growth stages of peanut plants. Irrigation was performed using a sprinkler irrigation system throughout both growing seasons. The first irrigation was applied at planting to ensure uniform germination. During the vegetative growth stage (approximately 15-30 days after emergence), irrigation was carried out once a week with approximately 30-40 mm of water. During the flowering stage (approximately 40-60 days after emergence), since this stage is particularly sensitive to water stress, the irrigation frequency was increased to twice a week with approximately 40-50 mm of water in each application. During the pod formation and pod filling stages (approximately 60-110 days after emergence), irrigation was continued twice a week with 40-50 mm of water to support pod development and seed filling. To facilitate pod maturation and reduce soil moisture for harvesting, irrigation was stopped approximately 15-20 days before harvest. Weed control was carried out mechanically between rows using a cultivator approximately 20 to 40 days after plant emergence, followed by manual weed removal as needed. Pest and disease management was performed based on regular field monitoring, and pesticide applications were made when pest or disease thresholds were exceeded, in accordance with integrated pest management principles. For planting preparation, the field was deeply plowed with a plow in the fall and mixed with a cultivator in the spring prior to planting; fertilizer was applied and mixed with a Goble disk to prepare the soil for planting. Planting was conducted on May 11 in the first year and on May 1 in the second year. During planting, peanut seeds were treated with a pesticide containing 80% Thiram active ingredient against crown rot (*Aspergillus niger*) and with a pesticide containing Endosulfan active ingredient against soil-borne pests. Harvesting was conducted manually on October 25 and October 29, respectively, in both years.

### 2.3 Sample collection and measurement

To determine the yield and yield components of peanut cultivars within the scope of this study, the following parameters were assessed: pod yield, first quality pod rate, Number of pods per plant, pod weight per plant, one hundred pod weight, one hundred seed weight and shelling ratio. First quality pod rate was determined as the percentage of well-filled, mature, and undamaged pods relative to the total number of harvested pods. Shelling ratio was calculated as the ratio of seed weight to total pod weight, expressed as a percentage. To determine quality and biochemical characteristics, oil content, protein content, fatty acid composition, iodine value, total antioxidant activity, total phenolic content, and total flavonoid content were analyzed (Beycioglu, 2022; Yılmaz et al., 2025).

Within the scope of physiological measurements, the leaf area index (LAI) and chlorophyll contents (total chlorophyll, chlorophyll-a, and chlorophyll-b) were determined during the plants' 50% flowering and pod formation stages. Chlorophyll measurements were conducted using a Falker CFL260 portable chlorophyll meter (Falker, Brazil), which provides relative chlorophyll index values ​​(CFL units) based on light transmittance at 635 nm and 880 nm wavelengths without requiring leaf extraction, at approximately 50–55 days after planting (corresponding to the 50% flowering stage) and at approximately 85–90 days after planting (corresponding to the pod formation stage). All measurements were taken between 08:00 and 10:00 a.m. to minimize the effects of diurnal variation on chlorophyll readings. Leaf Area Index (LAI) values ​​were determined using the BLAM-2 Portable Leaf Area Meter (Biobase, China) on the leaves of plants representing each plot.

### 2.4 Determination of total antioxidant activity (DPPH method)

The DPPH method described by Blasi et al. (2016) was used to determine antioxidant levels. Chemical Preparation: - Prepare 0.26 mM DPPH (2,2-diphenyl-1-picrylhydrazyl) (0.01025 g required per 100 mL, dissolved in ethyl alcohol). Blank or Control Preparation: 3000 μL (3 mL) of ethanol, 1000 μL (1 mL) of DPPH solution. Sample Preparation: Sample (taken from the stock solution) + ethanol = 3000 μL, 1000 μL (1 mL) of DPPH solution. After vortexing, the prepared samples were incubated in the dark for up to 30 minutes (readings were taken within 15 minutes after adding the DPPH solution). Subsequently, measurements were taken at a wavelength of 517 nm using a spectrophotometer.

### 2.5 Determination of total flavonoid content

The total flavonoid content was determined using a modified version of the method reported by Chang et al. (2002). Chemical Preparation: 10% Al(NO_3_)_3_·9H_2_O (aluminum nitrate) (10 g required for 100 mL, dissolved in methanol). 1 M CH_3_COONH_4_ (sodium acetate) (7.708 g required for 100 mL, dissolved in methanol). Blank or Control Preparation: 4300 μL (4.3 mL) methanol, 100 μL (0.1 mL) aluminum nitrate, 100 μL (0.1 mL) sodium acetate. Sample Preparation: Sample (taken from the stock solution) + methanol = 4300 μL (4.3 mL), 100 μL (0.1 mL) aluminum nitrate, 100 μL (0.1 mL) sodium acetate. After vortexing, the prepared samples were incubated in the dark for 40 minutes and then measured at a wavelength of 415 nm using a spectrophotometer. The total flavonoid content was expressed as mg quercetin equivalent (QE)/100 g extract.

### 2.6 Total phenolic content determination

The total phenolic content was determined using the modified Folin-Ciocalteu method (Singleton et al., 1999). According to this method, 6 mL of distilled water; 100 µL of blank, sample, or standard; 500 µL of Folin-Ciocalteu reagent; and 1500 µL of 20% sodium carbonate (Na_2_CO_3_) were added, and the final volume was adjusted to 10 mL with distilled water. After adding the Folin-Ciocalteu reagent, the mixture was allowed to stand for 5 minutes before adding the sodium carbonate. The prepared mixture was left to stand in the dark at room temperature for 2 hours, after which the absorbance of the blue solution was measured at 760 nm. The amount of phenolic compounds in the samples was calculated as the equivalent of gallic acid based on the standard calibration curve prepared using gallic acid.

### 2.7 Data analysis

The two-year data obtained from the study were subjected to analysis of variance (ANOVA) using JMP^®^ Pro 17.0 (SAS Institute Inc., 2021) statistical software. The statistical significance levels of the cultivar and year and the interactions between these factors (cultivar × year) were determined in the study. Differences between averages were evaluated using the Least Significant Difference (LSD) multivariate comparison test at the *p* < 0.05 significance level. Pearson correlation analysis was used to determine the two-variable relationships and distribution characteristics among the examined parameters. To determine the relationships between variables, a correlation matrix, heat map, and hierarchical clustering analyses were performed using the “ggplot2” (Wickham, 2016) package in the R Studio 2022.07.1 (RStudio, PBC) environment. The interpretation approach proposed by Kirca, (2025) was utilized for the joint assessment of yield and quality components. All graphical outputs and visualizations were created through the integration of JMP^®^ Pro and R software.

## 3 Results

### 3.1 Variance analysis and descriptive statistics

The descriptive statistical values for the agronomic, quality, and physiological characteristics examined in the study are presented in **Table 1**. Analysis of the data for the examined parameters revealed significant variation among the cultivars. Pod yield was found to range from 2556.30 to 5035.00 kg ha^-1^. Thus, it is evident that these cultivars possess high genetic diversity in terms of yield potential. Similarly, this situation was observed in key yield parameters such as hundred-pod weight (147.53–294.00 g) and hundred-seed weight (80.29–185.25 g). When quality characteristics were examined, it was found that oil content ranged from 46.07% to 58.21%, while protein content ranged from 22.12% to 29.49%. The most significant variation in fatty acid composition was observed in the ratios of oleic acid (41.11–73.51%) and linoleic acid (11.08–39.84%). This wide range of variation indicates that the peanut cultivars examined included both standard and high-oleic acid cultivars. Among the physiological parameters, the leaf area index (LAI) was determined to range from 4.32 to 10.30 during the flowering stage and from 4.07 to 11.71 during the pod development stage. This highlights the differences in the vegetative growth stages of the plants. When the coefficient of variation (CV%) values were examined, it was determined that the data generally had low CV values and that the experiment had high precision. The highest coefficients of variation were observed in chlorophyll-b (18.57% during the flowering period and 13.54% during the pod formation period) and the leaf area index (6.57–7.58). In contrast, very low CV% values were obtained for quality parameters such as phenolic content (0.18%), oleic acid (0.29%), linoleic acid (0.32%), and protein content (0.61%) (**Table 2**).

**Table 2:** Descriptive statistical values for the studied characteristics of peanut cultivars.

Variable	Abbreviation	Unit	Min	Max	Mean	StDev	CV%
Yield	Yield	kg ha^-1^	2556.30	5035.00	3746.10	757.60	2.02
First Quality Pod Rate	FiQuPoPe	%	52.06	79.08	61.39	5.94	2.26
Number of pods per plant	NuPoPePl	quantity	19.00	45.00	32.10	7.74	4.92
Weight of pods per plant	WePoPePl	g	39.58	64.30	51.42	6.14	2.86
One hundred pod weight	OhuPoWe	g	147.53	294.00	225.13	38.80	3.23
One hundred seed weight	OHuSeWe	g	80.29	185.25	132.39	26.39	4.59
Shelling ratio	SeRa	%	50.38	70.80	58.99	5.51	5.68
Oil content	Oc	%	46.07	58.21	51.83	2.68	2.01
Protein content	Pc	%	22.12	29.49	26.02	1.86	0.61
Leaf area index during flowering period	LeArInDuFlPe	-	4.32	10.30	6.85	1.63	6.57
Total chlorophyll amount during flowering period	TaChAmDuFlPe	CFL index	27.50	58.70	43.80	6.22	5.80
Chlorophyll a during flowering	ChADuFl	CFL index	21.00	39.00	28.89	2.71	5.67
Chlorophyll b during flowering	ChBDuFl	CFL index	5.00	25.00	14.16	4.36	18.57
Leaf area index during pod period	LeArInDuPoPe	-	4.07	11.71	7.13	1.84	7.58
Total chlorophyll in pod stage	ToChPoSt	CFL index	30.40	57.00	45.99	5.81	6.45
Chlorophyll a in pod period	ChAPoPe	CFL index	25.00	33.00	29.50	1.98	5.49
Chlorophyll b in pod period	ChBPoPe	CFL index	5.00	25.00	15.72	4.32	13.54
Palmitic acid	C16:0	%	6.45	10.48	9.42	0.94	0.84
Stearic acid	C18:0	%	1.48	2.98	2.19	0.47	0.45
Oleic acid	C18:1	%	41.11	73.51	48.14	7.51	0.29
Linoleic acid	C18:2	%	11.08	39.84	33.85	6.78	0.32
Arachidic acid	C20:0	%	0.80	1.40	1.07	0.16	0.93
Eicosenoic acid	C20:1	%	1.26	2.12	1.64	0.27	0.61
Behenic acid	C22:0	%	2.15	2.75	2.44	0.16	2.05
Lignoceric acid	C24:0	%	0.97	1.58	1.28	0.17	3.15
Iodine Value	IodVa	IV	82.42	105.14	100.04	5.33	0.30
Antioxidant Activity	AnOxDaAc	%	80.15	87.87	83.24	1.27	1.06
Phenolic Content	PheCo	mg GAE/100 g	175.36	254.78	215.24	22.29	0.18
Flavonoid Content	FlaCo	mg QE/100g	50.96	120.78	83.96	20.46	0.68

** indicates statistical significance at the *p* < 0.01 probability level.

* indicates statistical significance at the *p* < 0.05 probability level.

ns indicates a non-significant result (*p* > 0.05).

Small letters (a, b, c, …) following the mean values indicate the results of the LSD (Least Significant Difference) multiple comparison test; means sharing the same letter are not significantly different from each other at the *p* < 0.05 significance level.

### 3.2 Pod yield, yield components, and oil and protein content

The average values for pod yield, yield components, and oil and protein content of the peanut cultivars examined in the study, along with the results of the analysis of variance, are presented in **Table 3**. Cultivar (C), year (Y), and the C × Y interaction were found to be statistically significant at the *p* < 0.01 level for all parameters. Pod yield ranged from 2556.30 to 5035.00 kg ha⁻¹ across all cultivar × year interactions, with the highest yields obtained in Osmaniye 2005 (4815.70 kg ha⁻¹), Sultan (4683.50 kg ha⁻¹), and Halisbey (4649.70 kg ha⁻¹) cultivars. The lowest pod yield were observed in Batem Cihangir (2746.90 kg ha⁻¹) and Wilson (2885.20 kg ha⁻¹) cultivars. Considering the annual average, it was determined that pod yield for 2023 (3851.00 kg ha⁻¹) was higher than that for 2024 (3641.10 kg ha⁻¹). The C × Y interaction was found to be statistically significant (F = 11.79, *p* < 0.01). In 2023, the highest pod yield was recorded in the Osmaniye 2005 cultivar (4965.70 kg ha⁻¹, group a), while in 2024, the Osmaniye 2005 (4665.70 kg ha⁻¹, group bc) and Sultan (4446.00 kg ha⁻¹, group cd) cultivars came to the forefront. First quality pod rate ranged from 52.06% to 79.08%. Based on cultivar averages, the highest values were observed in the NC-7 (72.18%) and Brantley (70.91%) cultivars, both of which were placed in the same statistical group (group a). Although the year effect on this trait was statistically insignificant (F = 0.11, p > 0.05), the C × Y interaction was highly significant (F = 15.98, *p* < 0.01), indicating that cultivar performance varied across years. In 2023, the NC-7 cultivar recorded the highest first quality pod rate (75.67%, group a), while in 2024, the Brantley cultivar (71.62%, group b) ranked higher than NC-7 (68.69%, group c), suggesting that environmental conditions differentially affected cultivar performance for this trait. The number of pods per plant ranged from 19.00 to 45.00, with the highest values obtained in the Sultan (42.33) and Osmaniye 2005 (39.50) cultivars, and the lowest value recorded in the Wilson (21.83) cultivar. The 2023 growing season yielded a significantly higher number of pods per plant (34.23) compared to 2024 (29.97). The significant C × Y interaction (F = 4.65, *p* < 0.01) revealed that the Brantley cultivar achieved its highest pod number in 2023 (43.00, group a), while Sultan maintained consistently high values across both years (43.00 in 2023 and 41.67 in 2024, both group a), indicating greater stability for this trait.Pod weight per plant ranged from 39.58 to 64.30 g, with the highest values observed in the Osmaniye 2005 (59.25 g) and Brantley (58.93 g) cultivars. The year 2023 had a higher pod weight per plant (53.42 g) compared to 2024 (49.43 g). The C × Y interaction was found to be statistically significant (F = 8.78, *p* < 0.01). In 2023, the highest pod weight per plant was recorded in the Brantley cultivar (63.93 g, group a), while in 2024, the Osmaniye 2005 (58.59 g, group bc) and Sultan (52.37 g, group fg) cultivars stood out. The Brantley cultivar (53.93 g, group ef) had the lowest pod weight per plant in the same year.One hundred pod weight ranged from 147.53 to 294.00 g, with the Sultan (271.50 g) and Osmaniye 2005 (263.02 g) cultivars both falling into the highest-ranking group (group a) based on cultivar averages. However, due to the significant C × Y interaction, these cultivars showed divergent responses across years. Sultan and Osmaniye 2005 were grouped together in 2023 (282.00 g and 277.33 g, respectively, group a), whereas in 2024, Sultan (261.00 g, group b) ranked higher than Osmaniye 2005 (248.71 g, group d). The lowest value was observed in the Arıoğlu 2003 (156.62 g) cultivar."One hundred seed weight ranged from 80.29 to 185.25 g, with the highest values recorded in the Sultan (158.87 g), NC-7 (155.58 g), and Brantley (155.15 g) cultivars. The year 2023 had a higher one hundred seed weight (144.58 g) compared to 2024 (120.20 g). The C × Y interaction was found to be statistically significant (F = 4.08, *p* < 0.01). In 2023, the Brantley cultivar had the highest one hundred seed weight (172.41 g, group a), followed by NC-7 (171.03 g, group a). In 2024, higher values were observed in the Sultan (151.91 g, group cd) and Osmaniye 2005 (140.07 g, group e-g) cultivars.The shelling ratio varied between 50.38% and 70.80%, with the highest value observed in the Brantley cultivar (64.47%, group a) when considering the cultivar averages. This was followed by NC-7 (62.49%) and Wilson (61.44%). The lowest shelling ratio values were determined in Halisbey (54.84%) and Osmaniye 2005 (55.24%) cultivars. However, the statistically significant cultivar × year interaction (F = 21.87; *p* < 0.01) indicates that year-dependent differences should be included in the evaluation. When examining the data for 2023, Wilson (69.61%, group a) and NC-7 (68.21%, group ab) cultivars were ahead of Brantley (66.93%, group b). In 2024, the ranking changed. The Brantley cultivar (62.02%) showed superior performance compared to the NC-7 (56.76%) and Wilson (53.26%) cultivars. Oil content ranged from 46.07% to 58.21%, with the highest values ​​recorded in Brantley (54.95%) and Flower 22 (54.30%) varieties, and the lowest value in Osmaniye 2005 (48.95%).

**Table 3:** Average values for yield, first quality pod rate, number of pods per plant, weight of pods per plant, one hundred pod weight, one hundred seed weight, shelling ratio, oil content protein content and the resulting groups (cultivar, year and cultivar x year interaction).

*Cultivars*	Yield	FiQuPoRa	NuPoPePl	WePoPePl	OhuPoWe	OhuSeWe	SeRa	Oc	Pc
Arıoglu 2003 (1)	3350.50 ± 72.40^e^	56.06 ± 1.19 ^e^	23.50 ± 1.18 ^f^	44.68 ± 0.73 ^d^	156.62 ± 2.91 ^g^	87.05 ± 1.87 ^e^	55.58 ± 0.64 ^e^	50.14 ± 1.52 ^de^	27.28 ± 0.08 ^c^
Batem 5025 (2)	4187.50 ± 37.90^c^	57.50 ± 1.82 ^c-e^	27.50 ± 1.67 ^e^	49.42 ± 1.27 ^c^	237.54 ± 5.03 ^d^	143.67 ± 7.20 ^b^	60.30 ± 1.76 ^cd^	52.25 ± 0.25 ^b^	24.54 ± 0.20 ^h^
Batem Cihangir (3)	2746.90 ± 81.30^g^	61.29 ± 1.01 ^b^	23.17 ± 1.30 ^f^	51.00 ± 2.28 ^c^	205.33 ± 7.50 ^e^	115.94 ± 7.22 ^d^	56.20 ± 1.46 ^e^	51.90 ± 1.32 ^bc^	28.09 ± 0.61 ^a^
Brantley (4)	3469.90 ± 97.70^de^	70.91 ± 0.54 ^a^	38.50 ± 2.09 ^bc^	58.93 ± 2.24 ^a^	239.88 ± 8.56 ^cd^	155.15 ± 8.51 ^a^	64.47 ± 1.32 ^a^	54.95 ± 0.85 ^a^	25.55 ± 0.29 ^g^
Flower 22 (5)	3173.30 ± 62.40 ^f^	56.92 ± 0.87 ^de^	34.50 ± 1.67 ^d^	49.78 ± 0.67 ^c^	185.32 ± 2.13 ^f^	113.02 ± 6.57 ^d^	60.85 ± 2.97 ^bc^	54.30 ± 1.38 ^a^	22.88 ± 0.28 ^ı^
Halisbey (6)	4649.70 ± 51.70 ^b^	60.98 ± 0.56 ^b^	36.83 ± 0.60 ^c^	50.73 ± 0.58 ^c^	246.06 ± 9.59 ^bc^	135.08 ± 6.03 ^c^	54.84 ± 0.45 ^e^	52.17 ± 0.61 ^b^	27.09 ± 0.21 ^d^
NC-7 (7)	3498.70 ± 38.80 ^d^	72.18 ± 1.75 ^a^	33.33 ± 0.56 ^d^	54.23 ± 0.64 ^b^	253.84 ± 4.46 ^b^	155.58 ± 8.10 ^a^	62.49 ± 2.69 ^b^	51.51 ± 0.52 ^bc^	25.83 ± 0.83 ^f^
Osmaniye 2005 (8)	4815.70 ± 69.70 ^a^	59.06 ± 1.13 ^c^	39.50 ± 0.88 ^b^	59.25 ± 1.26 ^a^	263.02 ± 7.16 ^a^	145.01 ± 2.46 ^b^	55.24 ± 1.04 ^e^	48.95 ± 0.71 ^e^	25.41 ± 0.40 ^g^
Sultan (9)	4683.50 ± 114.10 ^ab^	58.12 ± 0.69 ^cd^	42.33 ± 0.71 ^a^	54.68 ± 1.14 ^b^	271.50 ± 5.56 ^a^	158.87 ± 4.04 ^a^	58.51 ± 0.82 ^d^	51.41 ± 0.47 ^bc^	26.07 ± 0.96 ^e^
Wilson (10)	2885.20 ± 28.40 ^g^	60.85 ± 0.23 ^b^	21.83 ± 0.87 ^f^	41.51 ± 0.49 ^e^	192.24 ± 1.98 ^f^	114.52 ± 7.43 ^d^	61.44 ± 3.68 ^bc^	50.74 ± 0.53 ^cd^	27.51 ± 0.24 ^b^
*Years*									
2023 (1)	3851.00 ± 140.90 ^a^	61.47± 1.23	34.23 ± 1.33 ^a^	53.42 ± 6.28 ^a^	236.08 ± 7.41 ^a^	144.58 ± 4.67 ^a^	62.07 ± 1.07 ^a^	52.16 ± 0.63	25.37 ± 0.28 ^b^
2024 (2)	3641.10 ± 135.30 ^b^	61.31± 0.94	29.97 ± 1.40 ^b^	49.43 ± 5.39 ^b^	214.18 ± 6.23 ^b^	120.20 ± 4.10 ^b^	55.92 ± 0.51 ^b^	51.50 ± 0.28	26.68 ± 0.35 ^a^
*C x Y*									
1-1	3499.30 ± 44.30 ^fg^	54.14 ± 1.82 ^ı^	26.00 ± 0,58 ^f^	46.01 ± 0.80 ^k^	161.76 ± 3.40 ^h^	90.92 ± 0.52 ^jk^	56.25 ± 1.24 ^f-ı^	46.86 ± 0.43 ^ı^	27.20 ± 0.16 ^ef^
1-2	3201.60 ± 45.80 ^ıj^	57.98 ± 0.28 ^gh^	21.00 ± 0,58 ^hı^	43.34 ± 0.48 ^l^	151.49 ± 2.12 ^h^	83.19 ± 1.52 ^k^	54.91 ± 0.24 ^h-j^	53.41 ± 0.78 ^bc^	27.37 ± 0.04 ^de^
2-1	4118.20 ± 43.90 ^e^	53.54 ± 0.63 ^ı^	31.00 ± 1,15 ^e^	52.17 ± 0.67 ^fg^	248.69 ± 1.35 ^dc^	159.75 ± 0.80 ^bc^	64.24 ± 0.14 ^c^	52.57 ± 0.09 ^cd^	24.25 ± 0.35 ^l^
2-2	4256.80 ± 21.80 ^de^	61.46 ± 0.76 ^d-f^	24.00 ± 0,58 ^fg^	46.67 ± 0.19 ^jk^	226.38 ± 0.70 ^e^	127.60 ± 0.66 ^ı^	56.36 ± 0.16 ^f-ı^	51.93 ± 0.45 ^c-e^	24.82 ± 0.02 ^j^
3-1	2928.00 ± 14.90 ^k^	59.21 ± 0.86 ^fg^	25.67 ± 0,88 ^fg^	56.00 ± 0.69 ^de^	222.04 ± 1.12 ^e^	132.00 ± 1.24 ^g-ı^	59.45 ± 0.30 ^de^	48.99 ± 0.38 ^gh^	26.73 ± 0.02 ^gh^
3-2	2565.80 ± 7.30 ^l^	63.37 ± 0.24 ^d^	20.67 ± 1,20 ^ı^	46.00 ± 0.69 ^k^	188.61 ± 1.00 ^fg^	99.87 ± 1.02 ^j^	52.95 ± 0.26 ^j^	54.81 ± 0.38 ^b^	29.45 ± 0.03 ^a^
4-1	3671.80 ± 79.20 ^f^	70.21 ± 0.94 ^bc^	43.00 ± 1,15 ^a^	63.93 ± 0.21 ^a^	257.33 ± 7.51 ^b-d^	172.41 ± 1.94 ^a^	66.93 ± 1.16 ^b^	56.59 ± 0.79 ^a^	24.91 ± 0.03 ^j^
4-2	3268.00 ± 27.40 ^h-j^	71.62 ± 0.25 ^b^	34.00 ± 0,58 ^cd^	53.93 ± 0.21 ^ef^	222.42 ± 2.24 ^e^	137.89 ± 1.15 ^f-h^	62.02 ± 1.14 ^cd^	53.31 ± 0.58 ^bc^	26.19 ± 0.03 ^ı^
5-1	3310.80 ± 16.70 ^g-ı^	58.09 ± 1.49 ^gh^	38.00 ± 1,15 ^b^	50.95 ± 0.84 ^g-ı^	189.33 ± 1.76 ^fg^	127.61 ± 0.87 ^hı^	67.41 ± 0.85 ^ab^	57.31 ± 0.58 ^a^	23.50 ± 0.03 ^n^
5-2	3035.80 ± 16.80 ^jk^	55.76 ± 0.47 ^hı^	31.00 ± 0,58 ^e^	48.62 ± 0.46 ^ıj^	181.30 ± 1.85 ^g^	98.43 ± 1.58 j	54.29 ± 0.45 ^h-j^	51.29 ± 0.39 ^d-f^	22.26 ± 0.08 °
6-1	4745.60 ± 50.50 ^ab^	61.98 ± 0.53 ^de^	37.33 ± 0,88 ^b^	51.94 ± 0.15 ^f-h^	265.33 ± 8.74 ^b^	147.46 ± 5.21 ^d-f^	55.57 ± 0.63 ^g-j^	52.08 ± 0.90 ^c-e^	26.62 ± 0.01 ^h^
6-2	4553.90 ± 40.10 ^bc^	59.98 ± 0.53 ^e-g^	36.33 ± 0,88 ^bc^	49.52 ± 0.46 ^hı^	226.79 ± 3.51 ^e^	122.71 ± 1.30 ^ı^	54.11 ± 0.26 ^ıj^	52.26 ± 1.04 ^cd^	27.56 ± 0.05 ^cd^
7-1	3525.80 ± 11.70 ^fg^	75.67 ± 1.75 ^a^	34.00 ± 0,58 ^cd^	54.06 ± 1.35 ^ef^	260.67 ± 6.96 ^bc^	171.03 ± 9.02 ^a^	68.21 ± 0.84 ^ab^	51.46 ± 0.51 ^de^	23.98 ± 0.00 ^lm^
7-2	3471.60 ± 81.60 ^f-h^	68,69 ± 0.23 ^c^	32.67 ± 0,88 ^de^	54.40 ± 0.50 ^ef^	247.01 ± 2.06 ^d^	140.14 ± 2.86 ^e-g^	56.76 ± 1.63 ^f-h^	51.56 ± 0.85 ^de^	27.68 ± 0.02 ^c^
8-1	4965.70 ± 26.90 ^a^	61.45 ± 0.43 ^d-f^	41.00 ± 1,15 ^a^	59.92 ± 2.04 ^b^	277.33 ± 7.05 ^a^	149.93 ± 2.46 ^c-e^	54.16 ± 2.04 ^h-j^	47.51 ± 0.65 ^hı^	24.53 ± 0.01 ^k^
8-2	4665.70 ± 32.80 ^bc^	56.67 ± 0.71 ^h^	38.00 ± 0,58 ^b^	58.59 ± 1.82 ^bc^	248.71 ± 1.19 ^d^	140.07 ± 0.17 ^e-g^	56.32 ± 0.20 ^f-ı^	50.38 ± 0.12 ^e-g^	26.30 ± 0.03 ^ı^
9-1	4921.00 ± 65.80 ^a^	59.44 ± 0.72 ^fg^	43.00 ± 1,15 ^a^	57.00 ± 0.77 ^cd^	282.00 ± 6.00 ^a^	165.82 ± 5.41 ^ab^	58.82 ± 1.75 ^ef^	51.90 ± 0.59 ^c-e^	23.92 ± 0.03 ^m^
9-2	4446.00 ± 65.80 ^cd^	56.80 ± 0.32 ^h^	41.67 ± 0,88 ^a^	52.37 ± 0.74 ^fg^	261.00 ± 2.89 ^b^	151.91 ± 1.93 ^cd^	58.21 ± 0.46 ^e-g^	50.92 ± 0.71 ^d-f^	28.23 ± 0.13 ^b^
10-1	2824.50 ± 10.90 ^k^	60.95 ± 0.50 ^ef^	23.33 ± 0,88 ^gh^	42.19 ± 0.03 ^lm^	196.33 ± 1.20 ^f^	128.83 ± 8.34 ^hı^	69.61 ± 0.80 ^a^	49.71 ± 0.57 ^fg^	28.05 ± 0.03 ^b^
10-2	2945.90 ± 15.10 ^k^	60.74 ± 0.02 ^ef^	20.33 ± 0,88 ^ı^	40.83 ± 0.86 ^m^	188.14 ± 1.14 ^fg^	100.22 ± 1.25 ^j^	53.26 ± 0.35 ^j^	51.77 ± 0.09 ^c-e^	26.97 ± 0.02 ^fg^
*ANOVA*									
F *_cultivars_*	625.47 ^**^	99.44^**^	130.73^**^	88.06^**^	164.93^**^	91.41^**^	27.66^**^	17.53^**^	554.66^**^
F *_years_*	276.34 ^**^	0.11 ^ns^	190.51^**^	81.66^**^	348.70^**^	73.73^**^	160.52^**^	4.16 ^ns^	796.97^**^
F *_cultivars x year_*	11.79 ^**^	15.98^**^	4.65^**^	8.78^**^	3.84^**^	4.08^**^	21.87^**^	20.28^**^	196.16^**^

** indicates statistical significance at the *p* < 0.01 probability level.

* indicates statistical significance at the *p* < 0.05 probability level.

ns indicates a non-significant result (*p* > 0.05).

Small letters (a, b, c, …) following the mean values indicate the results of the LSD (Least Significant Difference) multiple comparison test; means sharing the same letter are not significantly different from each other at the *p* < 0.05 significance level.

The effect of year on oil content was found to be statistically insignificant (F = 4.16, p > 0.05). However, the C × Y interaction was found to be statistically significant (F = 20.28, *p* < 0.01). In 2023, the Brantley variety had the highest oil content (56.59%, group a), followed by Flower 22 (57.31%, group a). In 2024, Batem Cihangir showed a significant increase (54.81%, group b) compared to its oil content in 2023 (48.99%, group gh).Protein content varied between 22.12% and 29.49%, with the highest values ​​obtained in Batem Cihangir (28.09%) and Wilson (27.51%) cultivars, while the lowest value was determined in Flower 22 (22.88%). The average protein content in 2024 (26.68%) was found to be statistically significantly higher compared to 2023 (25.37%). The cultivar × year interaction was highly significant with the highest F value among all parameters examined (F = 196.16; *p* < 0.01), revealing that the cultivars responded to environmental conditions in strongly different ways. The Batem Cihangir cultivar had the lowest values ​​in 2023 (26.73%, group gh) and reached the highest value in 2024 (29.45%, group a). The Flower 22 cultivar, however, showed the opposite trend, declining from its highest value in 2023 (23.50%, group n) to its lowest value in 2024 (22.26%, group o).

### 3.3 Physiological parameters: leaf area index and chlorophyll content

The mean values and analysis of variance (ANOVA) results for the leaf area index (LAI) and chlorophyll content during the flowering and pod formation periods are presented in **Table 4**. It was determined that the cultivar and C × Y interaction were significant at the *p* < 0.01 level for all physiological parameters. During the flowering period, LAI values ranged from 4.32 to 10.30; the highest values were observed in the Halisbey (8.51) and Osmaniye 2005 (7.88) cultivars, while the lowest value was recorded in the Batem Cihangir (4.92) cultivar. In terms of the year effect, the LAI value during the flowering period in 2024 (7.24) was statistically higher than that in 2023 (6.47). Total chlorophyll content during the flowering period ranged from 27.50 to 58.70 CFL index, with the highest value obtained in the Osmaniye 2005 (50.13 CFL index) cultivar; the year was determined to have no statistically significant effect on this characteristic (F = 0.89, *p* > 0.05). Chlorophyll-a values ranged from 21.00 to 39.00 CFL index, with the highest value recorded in the Osmaniye 2005 cultivar (31.67). When analyzed by year, this trait was found to be statistically insignificant (F = 3.71, *p* > 0.05). When chlorophyll-b values were examined, cultivar averages ranged from 5.00 to 25.00, with the highest value recorded in the Osmaniye 2005 cultivar (17.67). During the pod formation period, LAI values ranged from 4.07 to 11.71, with the highest values recorded in the Halisbey (8.92) and NC-7 (8.55) cultivars. It was determined that the LAI value during the pod formation period in 2024 (7.97) was higher than that in 2023 (6.29). During the pod formation period, total chlorophyll content ranged from 30.40 to 57.00 CFL index, with the highest value recorded in the Wilson cultivar (55.22); the effect of year on this trait was determined to be statistically insignificant (F = 1.55, *p* > 0.05). During the fruit development period, chlorophyll-a values ranged from 25.00 to 33.00, with the highest value obtained from the Wilson cultivar (32.25). The effect of year and the C × Y interaction were found to be statistically insignificant for this parameter. During the pod formation period, chlorophyll-b values ranged from 5.00 to 25.00, with the highest value observed in the Wilson cultivar (22.75), while the effect of year was determined to be statistically insignificant (F = 3.05, *p* > 0.05).

**Table 4:** Average values for leaf area index during flowering period, total chlorophyll amount during flowering period, chlorophyll a during flowering, chlorophyll b during flowering, leaf area index during pod period, total chlorophyll in pod period, chlorophyll a in pod period, chlorophyll b in pod period and the resulting groups (cultivar, year, cultivar x year interaction).

*Cultivars*	LeArLnDuFıPe	TaChAmDuFlPe	ChADuFl	ChBDuFl	LeArLnDuPoPe	TaChAmDuPoPe	ChAPoPe	ChBPoPe
Arıoglu 2003 (1)	7.66 ± 0.21 ^b^	44.40 ± 0.97 ^cd^	28.17 ± 0.31 ^d^	15.00 ± 0.77 ^a-c^	6.22 ± 0.83 ^e^	46.31 ± 2.24 ^bc^	29.00 ± 0.58 ^b-d^	16.42 ± 1.55 ^bc^
Batem 5025 (2)	5.69 ± 0.53 ^e^	42.40 ± 1.24 ^de^	28.17 ± 0.31 ^d^	13.50 ± 1.09 ^b-d^	6.39 ± 0.71 ^de^	44.23 ± 1.20 ^c^	29.75 ± 0.85 ^bc^	14.08 ± 0.88 ^c^
Batem Cihangir (3)	4.92 ± 0.14 ^f^	40.13 ± 1.36 ^e^	27.50 ± 0.76 ^de^	11.50 ± 1.06 ^de^	7.04 ± 0.81 ^c^	43.38 ± 1.39 ^c^	28.50 ± 0.41 ^cd^	14.00 ± 0.94 ^c^
Brantley (4)	6.07 ± 0.47 ^de^	46.78 ± 2.02 ^bc^	30.83 ± 0.40 ^ab^	15.00 ± 1.98 ^a-c^	5.92 ± 0.36 ^e^	36.88 ± 2.02 ^d^	27.50 ± 0.92 ^d^	8.67 ± 1.50 ^d^
Flower 22 (5)	6.64 ± 0.15 ^c^	44.70 ± 3.31 ^cd^	28.50 ± 1.06 ^cd^	15.50 ± 2.32 ^ab^	6.04 ± 0.30 ^e^	46.05 ± 2.02 ^bc^	29.00 ± 0.58 ^b-d^	16.08 ± 1.46 ^bc^
Halisbey (6)	8.51 ± 0.47 ^a^	36.77 ± 3.07 ^f^	25.67 ± 1.20 ^e^	10.33 ± 1.96 ^e^	8.92 ± 1.00 ^a^	46.08 ± 2.41 ^bc^	29.17 ± 0.65 ^b-d^	16.17 ± 1.64 ^bc^
NC-7 (7)	7.60 ± 0.83 ^b^	41.85 ± 2.00 ^de^	29.17 ± 1.28 ^b-d^	13.33 ± 1.78 ^b-e^	8.55 ± 0.54 ^a^	48.38 ± 1.87 ^b^	30.33 ± 0.71 ^bc^	17.00 ± 1.44 ^b^
Osmaniye 2005 (8)	7.88 ± 0.33 ^b^	50.13 ± 2.94 ^a^	31.67 ± 1.67 ^a^	17.67 ± 2.06 ^a^	6.99 ± 0.43 ^cd^	45.34 ± 1.17 ^bc^	29.08 ± 0.97 ^b-d^	15.42 ± 0.42 ^bc^
Sultan (9)	7.06 ± 1.19 ^c^	48.93 ± 1.89 ^ab^	30.17 ± 0.70 ^a-c^	17.83 ± 1.22 ^a^	7.87 ± 1.06 ^b^	48.03 ± 1.11 ^b^	30.42 ± 0.58 ^ab^	16.67 ± 1.08 ^b^
Wilson (10)	6.56 ± 0.27 ^cd^	41.87 ± 1.21 ^de^	29.08 ± 0.82 ^b-d^	11.92 ± 1.00 ^c-e^	7.36 ± 0.09 ^bc^	55.22 ± 0.45 ^a^	32.25 ± 0.31 ^a^	22.75 ± 0.73 ^a^
*Years*								
2023 (1)	6.47 ± 0.28 ^b^	44.25 ± 0.86	28.52 ± 0.28	15.15 ± 0.62	6.29 ± 1.75 ^b^	45.64 ± 1.26	29.45 ± 0.41	15.45 ± 0.97
2024 (2)	7.24 ± 0.30 ^a^	43.34 ± 1.37	29.27 ± 0.64	13.17 ± 0.91	7.97 ± 1.55 ^a^	46.34 ± 0.83	29.55 ± 0.31	16.00 ± 0.56
*C x Y*								
1-1	7.41 ± 0.33 ^d-f^	45.40 ± 1.31 ^c^	28.00 ± 0.58 ^de^	16.00 ± 1.00 ^b-d^	4.39 ± 0.22 ^k^	41.87 ± 1.87 ^gh^	28.00 ± 0.58 ^ef^	13.33 ± 1.33 ^d^
1-2	7.91 ± 0.23 ^cd^	43.40 ± 1.40 ^c-f^	28.33 ± 0.33 ^c-e^	14.00 ± 1.00 ^c-f^	8.05 ± 0.29 ^de^	50.75 ± 1.36 ^bc^	30.00 ± 0.58 ^a-e^	19.50 ± 0.87 ^b^
2-1	4.60 ± 0.01 ^kl^	43.87 ± 1.91 ^c-f^	28.33 ± 1.53 ^c-e^	14.67 ± 1.76 ^c-f^	4.93 ± 0.31 ^jk^	41.77 ± 0.81 ^gh^	29.00 ± 1.53 ^c-f^	12.67 ± 1.33 ^d^
2-2	6.77 ± 0.50 ^fg^	40.93 ± 1.37 ^d-g^	28.00 ± 0.58 ^de^	12.33 ± 1.20 ^d-f^	7.86 ± 0.53 ^e^	46.70 ± 0.69 ^c-f^	30.50 ± 0.87 ^a-e^	15.50 ± 0.29 ^cd^
3-1	4.66 ± 0.15 ^kl^	40.75 ± 2.28 ^e-g^	27.00 ± 0.87 ^e^	13.00 ± 1.73 ^d-f^	5.23 ± 0.15 ^ı-k^	43.05 ± 3.09 ^f-h^	28.50 ± 0.87 ^ef^	13.50 ± 2.02 ^d^
3-2	5.19 ± 0.12 ^jk^	39.50 ± 1.90 ^fg^	28.00 ± 1.53 ^de^	10.00 ± 0.58 ^fg^	8.84 ± 0.13 ^cd^	43.70 ± 0.29 ^f-h^	28.50 ± 0.29 ^ef^	14.50 ± 0.29 ^d^
4-1	5.10 ± 0.05 ^j-l^	50.77 ± 0.70 ^b^	31.00 ± 2.00 ^bc^	18.67 ± 0.67 ^a-c^	5.15 ± 0.19 ^ı-k^	33.30 ± 2.75 ^ı^	27.00 ± 2.00 ^f^	5.33 ± 0.33 ^e^
4-2	7.04 ± 0.38 ^e-g^	42.80 ± 2.00 ^c-f^	30.67 ± 0.67 ^b-d^	11.33 ± 2.40 ^d-f^	6.69 ± 0.15 ^fg^	40.45 ± 0.20 ^h^	28.00 ± 0.00 ^ef^	12.00 ± 0.00 ^d^
5-1	6.65 ± 0.24 ^gh^	51.97 ± 0.50 ^b^	30.67 ± 0.58 ^b-d^	20.67 ± 0.33 ^a^	5.49 ± 0.17 ^ıj^	42.85 ± 1.70 ^f-h^	28.00 ± 0.58 ^ef^	13.67 ± 0.88 ^d^
5-2	6.63 ± 0.24 ^gh^	37.43 ± 1.26 ^g^	26.33 ± 0.88 ^e^	10.33 ± 0.33 ^fg^	6.59 ± 0.34 ^f-h^	49.25 ± 2.68 ^c-e^	30.00 ± 0.58 ^a-e^	18.50 ± 2.02 ^bc^
6-1	7.73 ± 0.30 ^de^	42.93 ± 2.17 ^c-f^	28.00 ± 0.67 ^de^	14.00 ± 2.08 ^d-f^	6.70 ± 0.15 ^f-h^	50.80 ± 1.86 ^bc^	30.33 ± 0.67 ^a-e^	19.33 ± 1.45 ^b^
6-2	9.30 ± 0.62 ^ab^	30.60 ± 2.06 ^h^	23.33 ± 1.20 ^f^	6.67 ± 1.20 ^g^	11.13 ± 0.36 ^a^	41.35 ± 1.76 ^gh^	28.00 ± 0.58 ^ef^	13.00 ± 1.15 ^d^
7-1	5.75 ± 0.12 ^ıj^	38.03 ± 0.78 ^g^	27.33 ± 0.88 ^e^	13.00 ± 3.00 ^d-f^	7.49 ± 0.16 ^ef^	51.50 ± 2.77 ^a-c^	31.67 ± 0.88 ^a-c^	19.00 ± 2.52 ^b^
7-2	9.46 ± 0.16 ^a^	45.67 ± 2.22 ^c^	31.00 ± 1.73 ^bc^	13,67 ± 2.60 ^d-f^	9.61 ± 0.57 ^bc^	45.25 ± 0.03 ^e-h^	29.00 ± 0.00 ^d-f^	15.00 ± 0.00 ^cd^
8-1	7.17 ± 0.10 ^d-g^	43.80 ± 1.17 ^c-f^	28.67 ± 0.67 ^c-e^	14.67 ± 0.88 ^c-f^	6.05 ± 0.22 ^g-ı^	44.93 ± 1.11 ^e-h^	28.67 ± 0.67 ^d-f^	15.33 ± 0.88 ^cd^
8-2	8.58 ± 0.22 ^bc^	56.47 ± 1.27 ^a^	34.67 ± 2.18 ^a^	20.67 ± 3.38 ^ab^	7.92 ± 0.13 ^e^	45.75 ± 2.34 ^d-g^	29.50 ± 2.02 ^b-f^	15.50 ± 0.29 ^cd^
9-1	9.71 ± 0.20 ^a^	45.00 ± 0.83 ^cd^	28.67 ± 0.88 ^c-e^	15.33 ± 0.67 ^c-e^	10.02 ± 0.02 ^b^	50.27 ± 0.85 ^b-d^	31.33 ± 0.88 ^a-d^	18.33 ± 1.67 ^bc^
9-2	4.41 ± 0.06 ^l^	52.87 ± 1.34 ^ab^	31.67 ± 0.33 ^b^	20.33 ± 0.88 ^ab^	5.72 ± 1.01 ^h-j^	45.80 ± 0.69 ^d-g^	29.50 ± 0.29 ^b-f^	15.00 ± 0.58 ^cd^
10-1	6.00 ± 0.08 ^hı^	40.00 ± 0.23 ^e-g^	27.50 ± 0.58 ^e^	11.50 ± 0.29 ^ef^	7.47 ± 0.16 ^ef^	56.04 ± 0.58 ^a^	32.00 ± 0.58 ^ab^	24.00 ± 1.00 ^a^
10-2	7.14 ± 0.05 ^e-g^	43.73 ± 1.94 ^c-e^	30.67 ± 0.88 ^b-d^	12.33 ± 2.19 ^d-f^	7.26 ± 0.09 ^ef^	54.40 ± 0.06 ^ab^	32.50 ± 0.29 ^a^	21.50 ± 0.29 ^ab^
*ANOVA*								
F *_cultivars_*	35.01^**^	15.36^**^	6.59^**^	5.44^**^	22.22^**^	14.17^**^	3.78^**^	15.82^**^
F *_years_*	62.26^**^	0.89^ns^	3.71^ns^	2.73^ns^	46.69^**^	1.55^ns^	0.13^ns^	3.05^ns^
F *_cultivars x year_*	40.73^**^	18.74^**^	6.45^**^	6.11^**^	31.90^**^	6.43^**^	1.77^ns^	6.77^**^

** indicates statistical significance at the *p* < 0.01 probability level.

* indicates statistical significance at the *p* < 0.05 probability level.

ns indicates a non-significant result (*p* > 0.05).

Small letters (a, b, c, …) following the mean values indicate the results of the LSD (Least Significant Difference) multiple comparison test; means sharing the same letter are not significantly different from each other at the *p* < 0.05 significance level.

### 3.4 Fatty acid composition, iodine value, antioxidant activity, and bioactive compounds

The mean values for fatty acid composition, iodine value, antioxidant activity, phenolic content, and flavonoid content of the cultivars, along with the results of the analysis of variance, are presented in **Table 5**. It was determined that the effects of cultivar, year and the C × Y interaction were significant at the *p* < 0.01 level for all parameters. The palmitic acid content ranged from 6.45% to 10.48%; the highest value was observed in the Batem Cihangir cultivar (10.39%), while the lowest value was observed in the Brantley cultivar (6.88%). It was found that the year effect was statistically insignificant for this parameter (F = 0.37, *p* > 0.05). The stearic acid content ranged from 1.48% to 2.98%, with the highest value obtained in the Wilson cultivar (2.67%). The oleic acid content showed a wide variation ranging from 41.11% to 73.51%. Among the cultivars, the Brantley cultivar had the highest value (68.44%), followed by the NC-7 (50.68%) and Wilson (47.88%) cultivars. Additionally, the lowest value was observed in the Batem Cihangir cultivar (41.68%). The average linoleic acid content among the cultivars ranged from 11.08% to 39.84%, with the highest value recorded in the Batem Cihangir cultivar (39.35%) and the lowest in the Brantley cultivar (15.73%). According to cultivar averages, the arachidic acid content ranged from 0.80% to 1.40%, the eicosenoic acid content from 1.26% to 2.12%, the behenic acid content from 2.15% to 2.75%, and the lignoseric acid content from 0.97% to 1.58%. Regarding the iodine value, the cultivar averages ranged from 82.42 to 105.14 IV. The highest values were observed in the Flower 22 (104.12 IV), Arıoğlu 2003 (104.05 IV), and Batem Cihangir (104.00 IV) cultivars, while the lowest value was observed in the Brantley (86.11 IV) cultivar. It was determined that the iodine value in 2023 (100.35 IV) belonged to a statistically different group compared to 2024 (99.73 IV) and had a higher value. Biochemical analysis revealed that total antioxidant activity ranged from 80.15% to 87.87% across the cultivar averages. The highest value was observed in the Flower 22 cultivar (84.26%). Total phenolic content ranged from 175.36 to 254.78 mg GAE/100 g. The highest total phenolic content was obtained in the Wilson (254.58 mg GAE/100 g) and Halisbey (245.76 mg GAE/100 g) cultivars. The lowest value was determined in the Brantley cultivar (179.08 mg GAE/100 g). When examined in terms of total flavonoid content, cultivar averages ranged from 50.96 to 120.78 mg QE/100 g, with the highest value determined in the Arıoğlu 2003 cultivar. This cultivar was followed by the NC-7 (105.49 mg QE/100 g) and Osmaniye 2005 (104.66 mg QE/100 g) cultivars. The lowest value was determined in the Brantley cultivar (53.38 mg QE/100 g). In 2024, the flavonoid content (85.00 mg QE/100 g) was found to be statistically significantly higher than in 2023 (83.10 mg QE/100 g).

**Table 5:** Average values for palmitic acid, stearic acid, oleic acid, linoleic acid, arachidic acid, eicosenoic acid, behenic acid, lignoseric acid, iodine value, antioxidant activity, phenolic content, flavonoid content and the resulting groups (cultivar, year, cultivar x year interaction).

*Cultivars*	C16:0	C18:0	C18:1	C18:2	C20:0	C20:1	C22:0	C24:0	IodVa	AnOxDaAc	PheCo	FlaCo
Arıoglu 2003 (1)	10.01 ± 0.08 ^b^	1.58 ± 0.02 ^h^	44.12 ± 0.13 ^h^	38.16 ± 0.09 ^c^	0.84 ± 0.01 ^ı^	1.75 ± 0.03 ^d^	2.33 ± 0.05 ^de^	1.37 ± 0.05 ^c^	104.05 ± 0.27 ^a^	82.34 ± 0.66 ^c^	206.20 ± 0.52 ^g^	119.63 ± 0.42 ^a^
Batem 5025 (2)	9.83 ± 0.14 ^c^	2.64 ± 0.09 ^b^	48.96 ± 0.05 ^c^	32.54 ± 0.05 ^h^	1.23 ± 0.03 ^b^	1.29 ± 0.01 ^h^	2.38 ± 0.07 ^cd^	1.12 ± 0.03 ^f^	98.48 ± 0.06 ^f^	83.07 ± 0.24 ^bc^	233.46 ± 0.59 ^c^	74.11 ± 1.08 ^f^
Batem Cihangir (3)	10.39 ± 0.03 ^a^	2.48 ± 0.13 ^d^	41.68 ± 0.20 ^j^	39.35 ± 0.19 ^a^	1.07 ± 0.02 ^e^	1.41 ± 0.05 ^g^	2.34 ± 0.07 ^de^	1.26 ± 0.03 ^d^	104.00 ± 0.16 ^a^	83.31 ± 0.20 ^a-c^	194.10 ± 0.71 ^ı^	67.97 ± 1.52 ^g^
Brantley (4)	6.88 ± 0.19 ^h^	2.61 ± 0.14 ^c^	68.44 ± 2.22 ^a^	15.73 ± 2.05 ^j^	1.21 ± 0.04 ^b^	1.79 ± 0.07 ^c^	2.28 ± 0.05 ^e^	1.05 ± 0.03 ^g^	86.11 ± 1.64 ^g^	82.41 ± 0.56 ^c^	179.08 ± 1.50 ^j^	53.38 ± 0.92 ^ı^
Flower 22 (5)	9.67 ± 0.02 ^d^	1.50 ± 0.00 ^ı^	43.04 ± 0.01 ^ı^	38.74 ± 0.00 ^b^	0.86 ± 0.00 ^h^	2.10 ± 0.00 ^a^	2.52 ± 0.02 ^b^	1.56 ± 0.01 ^a^	104.12 ± 0.01 ^a^	84.26 ± 0.84 ^a^	217.19 ± 0.59 ^d^	81.18 ± 2.16 ^e^
Halisbey (6)	9.60 ± 0.10 ^de^	1.92 ± 0.02 ^g^	45.29 ± 0.27 ^f^	36.35 ± 0.28 ^e^	0.95 ± 0.01 ^g^	1.81 ± 0.09 ^c^	2.61 ± 0.02 ^a^	1.46 ± 0.01 ^b^	101.92 ± 0.26 ^c^	83.81 ± 0.40 ^ab^	245.76 ± 0.32 ^b^	88.10 ± 1.67 ^d^
NC-7 (7)	9.16 ± 0.10 ^g^	2.44 ± 0.02 ^e^	50.68 ± 0.06 ^b^	31.57 ± 0.05 ^ı^	1.14 ± 0.00 ^c^	1.45 ± 0.05 ^f^	2.40 ± 0.04 ^cd^	1.15 ± 0.03 ^ef^	98.28 ± 0.04 ^f^	83.92 ± 0.56 ^ab^	208.05 ± 0.60 ^f^	105.49 ± 0.49 ^b^
Osmaniye 2005 (8)	9.43 ± 0.02 ^f^	1.91 ± 0.10 ^g^	44.34 ± 0.60 ^g^	37.52 ± 0.56 ^d^	1.02 ± 0.04 ^f^	1.84 ± 0.08 ^b^	2.55 ± 0.02 ^ab^	1.40 ± 0.06 ^c^	103.12 ± 0.46 ^b^	82.69 ± 0.45 ^c^	204.41 ± 0.39 ^h^	104.66 ± 3.59 ^c^
Sultan (9)	9.70 ± 0.10 ^d^	2.21 ± 0.01 ^f^	47.01 ± 0.24 ^e^	35.06 ± 0.16 ^f^	1.11 ± 0.02 ^d^	1.54 ± 0.02 ^e^	2.44 ± 0.08 ^c^	1.24 ± 0.02 ^d^	101.15 ± 0.32 ^d^	83.36 ± 0.30 ^a-c^	209.54 ± 1.49 ^e^	81.58 ± 3.46 ^e^
Wilson (10)	9.52 ± 0.05 ^ef^	2.67 ± 0.14 ^a^	47.88 ± 0.03 ^d^	33.49 ± 0.20 ^g^	1.27 ± 0.05 ^a^	1.45 ± 0.02 ^f^	2.54 ± 0.08 ^ab^	1.18 ± 0.02 ^e^	99.19 ± 0.36 ^e^	83.24 ± 0.31 ^a-c^	254.58 ± 0.07 ^a^	64.39 ± 1.33 ^h^
*Years*												
2023 (1)	9.41 ± 0.15	2.16 ± 0.08 ^b^	47.68 ± 1.10 ^b^	34.26 ± 0.99 ^a^	1.07 ± 0.03	1.70 ± 0.04 ^a^	2.49 ± 0.03 ^a^	1.28 ± 0.03	100.35 ± 0.79 ^a^	83.56 ± 0.27	215.38 ± 4.22	83.10 ± 3.62 ^b^
2024 (2)	9.43 ± 0.19	2.23 ± 0.09 ^a^	48.61 ± 1.61 ^a^	33.44 ± 1.46 ^b^	1.07 ± 0.03	1.58 ± 0.05 ^b^	2.38 ± 0.03 ^b^	1.28 ± 0.04	99.73 ± 1.14 ^b^	82.93 ± 0.16	215.09 ± 3.98	85.00 ± 3.94 ^a^
*C x Y*												
1-1	9.86 ± 0.08 ^d^	1.54 ± 0.01 ^q^	44.35 ± 0.16 ^k^	38.31 ± 0.13 ^d^	0.82 ± 0.01 ^l^	1.81 ± 0.02 ^d^	2.37 ± 0.10 ^fg^	1.29 ± 0.09 ^ef^	104.49 ± 0.37 ^a^	83.55 ± 0.09 ^b-d^	205.09 ± 0.30 ^l^	118.73 ± 0.22 ^b^
1-2	10.16 ± 0.01 ^c^	1.63 ± 0.00 ^p^	43.90 ± 0.08 ^l^	38.01 ± 0.04 ^e^	0.85 ± 0.02 ^kl^	1.70 ± 0.02 ^e^	2.29 ± 0.05 ^f-ı^	1.45 ± 0.01 ^cd^	103.60 ± 0.14 ^c^	81.13 ± 0.84 ^g^	207.31 ± 0.13 ^j^	120.53 ± 0.20 ^a^
2-1	10.15 ± 0.07 ^c^	2.45 ± 0.00 ^f^	49.07 ± 0.04 ^e^	32.44 ± 0.04 ^n^	1.15 ± 0.00 ^cd^	1.33 ± 0.01 ^l^	2.23 ± 0.00 ^e-j^	1.19 ± 0.01 ^h-j^	98.39 ± 0.10 ^g^	83.31 ± 0.45 ^cd^	234.65 ± 0.58 ^d^	71.74 ± 0.39 ^m^
2-2	9.52 ± 0.00 ^g-ı^	2.83 ± 0.01 ^c^	48.86 ± 0.03 ^e^	32.64 ± 0.02 ^m^	1.30 ± 0.00 ^b^	1.26 ± 0.00 ^m^	2.53 ± 0.03 ^cd^	1.05 ± 0.02 ^lm^	98.56 ± 0.06 ^g^	82.84 ± 0.18 ^d-f^	232.27 ± 0.10 ^e^	76.48 ± 0.29 ^k^
3-1	10.46 ± 0.01 ^a^	2.19 ± 0.01 ^k^	41.24 ± 0.07 °	39.77 ± 0.04 ^a^	1.03 ± 0.00 ^h^	1.53 ± 0.01 ^ı^	2.48 ± 0.05 ^de^	1.31 ± 0.05 ^e^	104.35 ± 0.13 ^a^	83.42 ± 0.24 ^cd^	195.65 ± 0.35 ^n^	71.32 ± 0.13 ^m^
3-2	10.32 ± 0.01 ^b^	2.78 ± 0.00 ^d^	42.13 ± 0.03 ^n^	38.93 ± 0.02 ^b^	1.12 ± 0.01 ^ef^	1.29 ± 0.01 ^m^	2.20 ± 0.01 ^ıj^	1.21 ± 0.02 ^f-h^	103.66 ± 0.02 ^bc^	83.21 ± 0.36 ^c-f^	192.55 ± 0.07 °	64.62 ± 0.59 °
4-1	7.31 ± 0.00 ^l^	2.29 ± 0.00 ^ı^	63.48 ± 0.06 ^b^	20.31 ± 0.03 ^q^	1.13 ± 0.01 ^de^	1.96 ± 0.01 ^c^	2.39 ± 0.02 ^ef^	1.12 ± 0.01 ^j-l^	89.78 ± 0.06 ^h^	81.66 ± 0.77 ^fg^	175.73 ± 0.33 ^q^	55.43 ± 0.19 ^q^
4-2	6.45 ± 0.00 ^m^	2.92 ± 0.01 ^b^	73.39 ± 0.07 ^a^	11.15 ± 0.04 ^r^	1.29 ± 0.00 ^b^	1.63 ± 0.00 ^fg^	2.18 ± 0.01 ^j^	0.97 ± 0.00 ^m^	82.44 ± 0.01 ^ı^	83.16 ± 0.64 ^c-f^	182.43 ± 0.03 ^p^	51.33 ± 0.29 ^r^
5-1	9.67 ± 0.02 ^ef^	1.50 ± 0.01 ^r^	43.04 ± 0.01 ^m^	38.74 ± 0.01 ^bc^	0.86 ± 0.00 ^k^	2.11 ± 0.01 ^a^	2.52 ± 0.01 ^cd^	1.56 ± 0.01 ^a^	104.11 ± 0.00 ^a-c^	85.45 ± 1.45 ^a^	218.51 ± 0.07 ^f^	76.35 ± 0.10 ^k^
5-2	9.68 ± 0.04 ^e^	1.49 ± 0.01 ^r^	43.05 ± 0.01 ^m^	38.74 ± 0.01 ^c^	0.85 ± 0.00 ^kl^	2.09 ± 0.01 ^a^	2.52 ± 0.03 ^cd^	1.56 ± 0.01 ^ab^	104.13 ± 0.02 ^ab^	83.06 ± 0.16 ^c-f^	215.87 ± 0.13 ^g^	86.00 ± 0.47 ^ı^
6-1	9.38 ± 0.03 ^j^	1.87 ± 0.01 ^n^	44.68 ± 0.02 ^j^	36.98 ± 0.02 ^f^	0.94 ± 0.01 ^j^	2.01 ± 0.01 ^b^	2.66 ± 0.02 ^ab^	1.48 ± 0.01 ^b-d^	102.49 ± 0.04 ^d^	84.61 ± 0.38 ^a-c^	246.44 ± 0.17 ^b^	84.38 ± 0.20 ^j^
6-2	9.83 ± 0.01 ^d^	1.96 ± 0.00 ^m^	45.89 ± 0.00 ^ı^	35.72 ± 0.01 ^h^	0.97 ± 0.00 ^ı^	1.61 ± 0.00 ^g^	2.57 ± 0.01 ^b-d^	1.44 ± 0.01 ^d^	101.35 ± 0.03 ^e^	83.01 ± 0.16 ^d-f^	245.09 ± 0.16 ^c^	91.82 ± 0.41 ^g^
7-1	8.94 ± 0.01 ^k^	2.40 ± 0.01 ^g^	50.81 ± 0.02 ^c^	31.46 ± 0.01 ^p^	1.13 ± 0.00 ^de^	1.57 ± 0.01 ^h^	2.48 ± 0.01 ^de^	1.21 ± 0.01 ^g-ı^	98.20 ± 0.01 ^g^	84.94 ± 0.70 ^ab^	206.74 ± 0.20 ^jk^	106.36 ± 0.31 ^d^
7-2	9.39 ± 0.01 ^ıj^	2.49 ± 0.00 ^e^	50.55 ± 0.01 ^d^	31.68 ± 0.01 °	1.14 ± 0.00 ^c-e^	1.34 ± 0.01 ^l^	2.31 ± 0.00 ^f-h^	1.09 ± 0.00 ^kl^	98.36 ± 0.03 ^g^	82.89 ± 0.25 ^d-f^	209.36 ± 0.30 ^ı^	104.62 ± 0.61 ^e^
8-1	9.40 ± 0.04 ^ıj^	2.13 ± 0.01 ^l^	45.67 ± 0.01 ^ı^	36.26 ± 0.03 ^g^	1.10 ± 0.01 ^f^	1.66 ± 0.01 ^f^	2.50 ± 0.03 ^cd^	1.27 ± 0.03 ^e-g^	102.09 ± 0.07 ^d^	81.80 ± 0.35 ^e-g^	203.57 ± 0.14 ^m^	96.63 ± 0.28 ^f^
8-2	9.46 ± 0.01 ^h-j^	1.69 ± 0.01 °	43.00 ± 0.00 ^m^	38.77 ± 0.01 ^bc^	0.94 ± 0.01 ^ıj^	2.02 ± 0.00 ^b^	2.59 ± 0.01 ^bc^	1.52 ± 0.02 ^a-c^	104.14 ± 0.03 ^ab^	83.58 ± 0.32 ^b-d^	205.25 ± 0.24 ^l^	112.69 ± 0.22 ^c^
9-1	9.54 ± 0.06 ^f-h^	2.24 ± 0.01 ^j^	46.58 ± 0.24 ^h^	35.30 ± 0.19 ^ı^	1.15 ± 0.01 ^cd^	1.58 ± 0.01 ^h^	2.60 ± 0.02 ^bc^	1.24 ± 0.01 ^e-h^	101.20 ± 0.54 ^e^	83.20 ± 0.23 ^c-e^	212.87 ± 0.11 ^h^	88.64 ± 0.09 ^h^
9-2	9.85 ± 0.16 ^d^	2.18 ± 0.01 ^k^	47.44 ± 0.20 ^g^	34.81 ± 0.17 ^j^	1.06 ± 0.01 ^g^	1.50 ± 0.02 ^j^	2.28 ± 0.07 ^g-ı^	1.25 ± 0.05 ^e-h^	101.10 ± 0.47 ^e^	83.53 ± 0.62 ^b-d^	206.20 ± 0.30 ^k^	74.52 ± 0.40 ^l^
10-1	9.42 ± 0.02 ^h-j^	2.97 ± 0.01 ^a^	47.84 ± 0.05 ^f^	33.04 ± 0.01 ^l^	1.38 ± 0.01 ^a^	1.48 ± 0.00 ^j^	2.73 ± 0.02 ^a^	1.13 ± 0.01 ^ı-k^	98.38 ± 0.06 ^g^	83.58 ± 0.58 ^b-d^	254.55 ± 0.12 ^a^	61.43 ± 0.32 ^p^
10-2	9.61 ± 0.02 ^e-g^	2.37 ± 0.00 ^h^	47.92 ± 0.01 ^f^	33.93 ± 0.02 ^k^	1.16 ± 0.01 ^c^	1.41 ± 0.01 ^k^	2.36 ± 0.01 ^fg^	1.23 ± 0.02 ^e-h^	99.99 ± 0.04 ^f^	82.90 ± 0.21 ^d-f^	254.60± 0.12 ^a^	67.36 ± 0.12 ^n^
ANOVA												
F *_cultivars_*	817.79^**^	6460.00^**^	17479.05^**^	22006.88^**^	552.14^**^	1092.43^**^	20.46^**^	70.44^**^	1843.33^**^	3.15^**^	22015.11^**^	7773.29^**^
F *_years_*	0.37^ns^	2476.53^**^	299.36^**^	338.92^**^	0.04	338.80^**^	27.24^**^	0.05^ns^	25.25^**^	2.68^ns^	3.30^ns^	138.64^**^
F *_cultivars x year_*	44.41^**^	1317.77^**^	827.89^**^	1113.47^**^	92.57^**^	188.54^**^	17.01^**^	11.66^**^	104.18^**^	4.43^**^	144.32^**^	321.26^**^

** indicates statistical significance at the *p* < 0.01 probability level.

* indicates statistical significance at the *p* < 0.05 probability level.

ns indicates a non-significant result (*p* > 0.05).

Small letters (a, b, c, …) following the mean values indicate the results of the LSD (Least Significant Difference) multiple comparison test; means sharing the same letter are not significantly different from each other at the *p* < 0.05 significance level.

### 3.5 Relationships between parameters and correlation analysis

**Figure 3:**
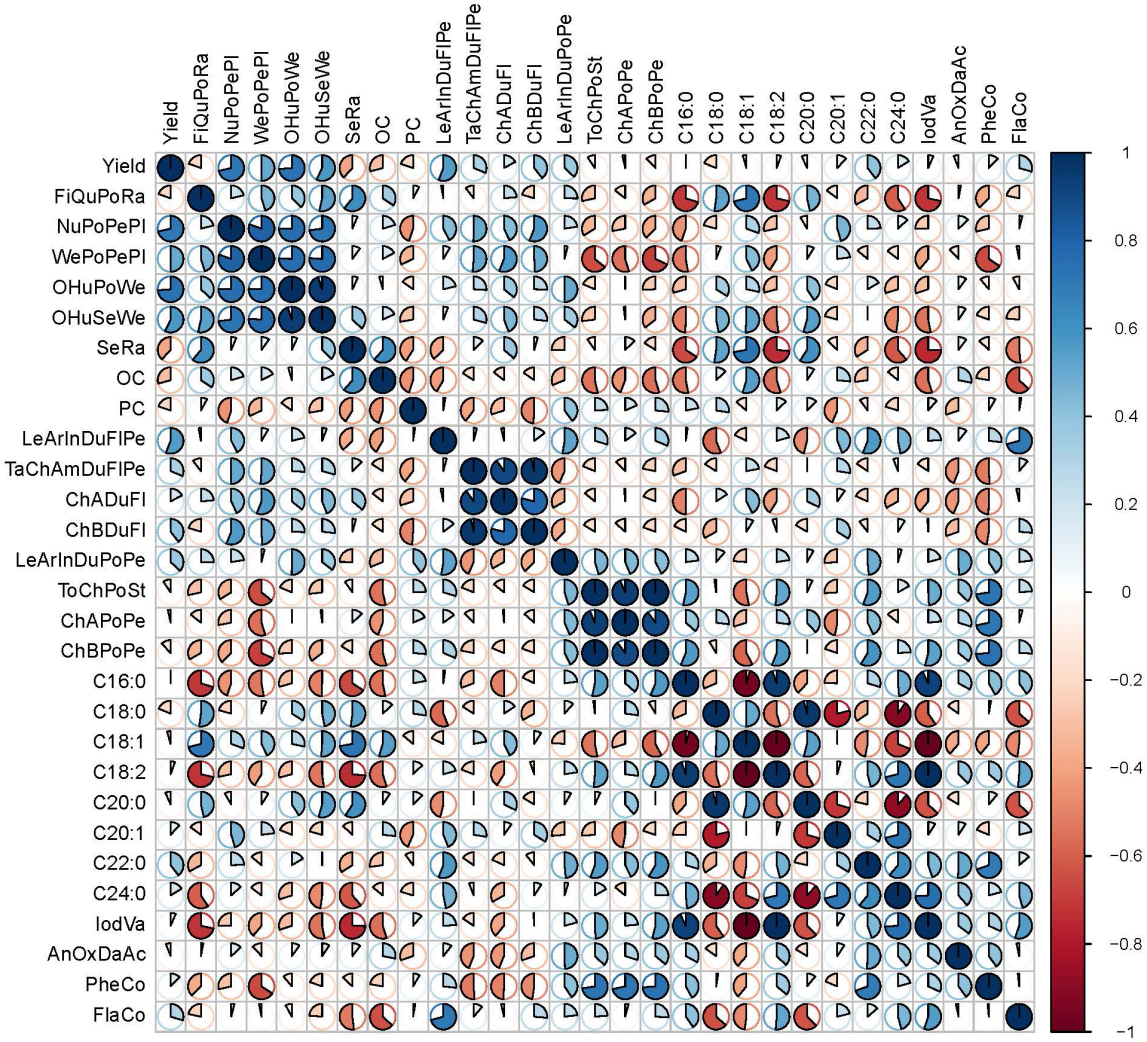
Correlation matrix of yield, leaf area index, chlorophyll content, fatty acid profile and bioactive compounds (The average of 2023 and 2024 years). Pearson correlation type was used in the correlation matrix analysis.

The results of the correlation analysis to determine the relationships among the agronomic, quality, and physiological characteristics of peanut cultivars are given in **Figure 3**. Analysis of the correlation matrix indicated statistically significant and positive correlations between pod yield (Yield) and the number of pod per plant (NuPoPePl), pod weight per plant (WePoPePl), hundred-pod weight (OHuPoWe), and hundred-seed weight (OHuSeWe). When examining the relationships between physiological parameters and yield, a positive correlation was found between the leaf area index (LAI) and chlorophyll contents (TaChAm, ChA, ChB) during the flowering and pod formation periods and the pod yield and pod weight parameters. In particular, it was observed that chlorophyll contents (ToChPoSt, ChAPoPe, ChBPoPe) during the pod formation period had a more pronounced positive effect on yield components compared to the flowering period. The relationships among fatty acid compositions were fully consistent with peanut biochemistry. Additionally, negative correlations were identified between oleic acid (C18:1) and linoleic acid (C18:2) as well as palmitic acid (C16:0). Similarly, a negative relationship was observed between the iodine value (IodVa) and oleic acid, while a positive relationship was noted with linoleic acid. In terms of bioactive compounds, it was determined that the total phenolic content (PheCo) showed a positive correlation with chlorophyll parameters during the pod development stage. Additionally, positive correlations were found between total flavonoid (FlaCo) content and the first quality pod ratio (FiQuPoRa) and the shelling ratio (SeRa). Positive correlations were observed between oil content (OC) and certain saturated fatty acids (C20:0, C22:0, C24:0) (**Figure 3**).

### 3.6 Heatmap and cluster analysis

**Figure 4:**
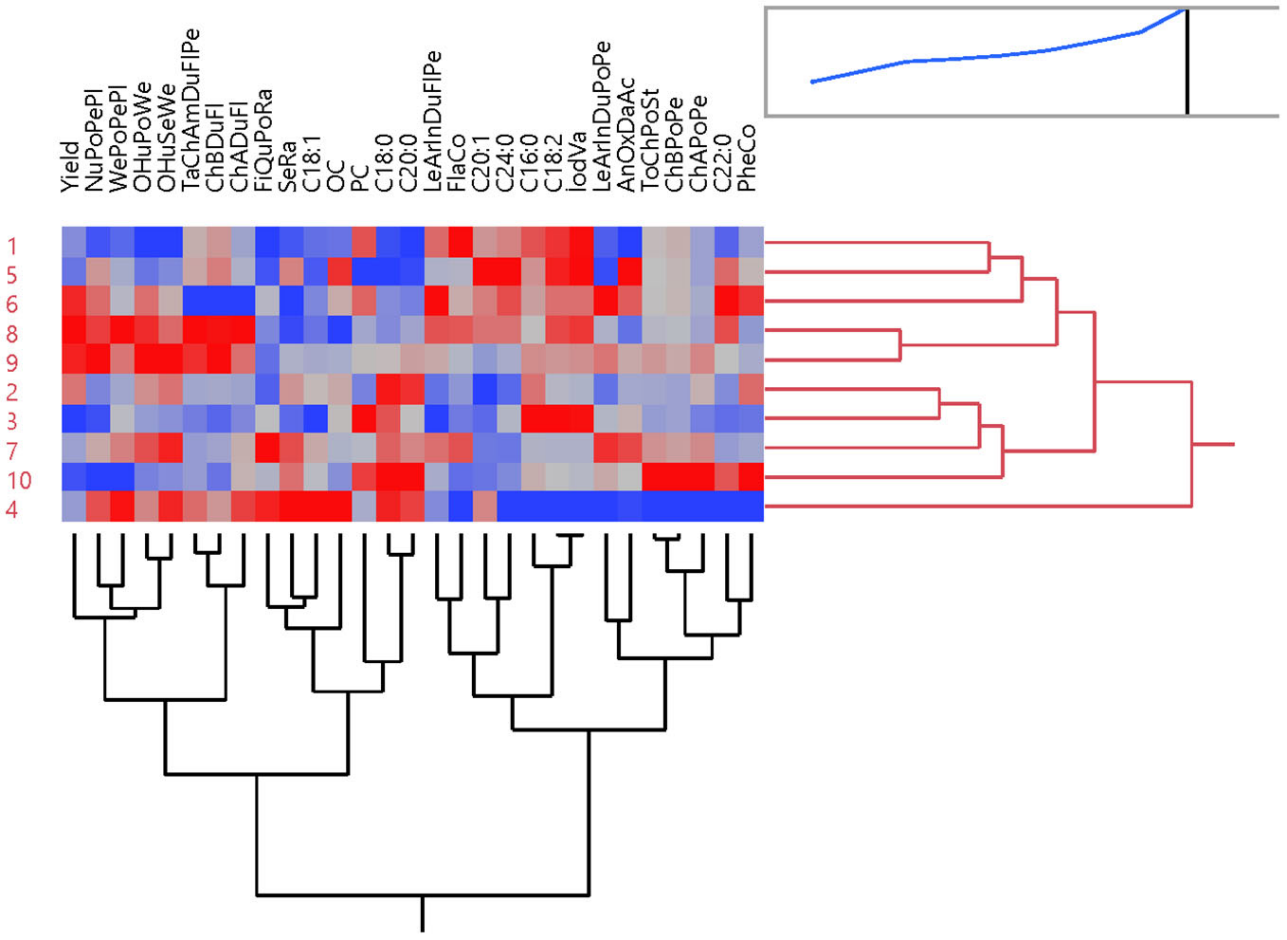
Heat map obtained as an output of the hierarchical cluster analysis of cultivars and traits studied.

The results of the heat map and hierarchical clustering analysis, conducted to identify the similarities and differences among peanut cultivars in terms of all agronomic, quality, and physiological traits examined, are presented in **Figure 4**. According to the dendrogram analysis, the cultivars were grouped into major clusters based on their characteristics. Upon examining the heat map, it was observed that the Brantley cultivar was distinctly separated from all other cultivars and formed a single main branch. It was determined that this cultivar exhibited the highest performance (dark blue color) particularly in the parameters of oleic acid, oil content, and eicosenoic acid, whereas it had the lowest values for linoleic acid (C18:2), palmitic acid (C16:0), and iodine value (IodVa). This indicates that the Brantley cultivar is the most prominent genetic material in the population due to its high oleic acid content. When evaluated in terms of yield and yield components, it was determined that the Osmaniye 2005, Sultan, and Halisbey cultivars form a cluster with similar characteristics. It was observed that these cultivars exhibited high performance in key yield parameters such as pod yield (Yield), number of pod per plant (NuPoPePl), and pod weight (WePoPePl). In particular, Osmaniye 2005 and Sultan cultivars are shown in the dendrogram to be the closest to each other in terms of yield potential. When examined in terms of physiological and bioactive properties, Wilson cultivar was observed to stand out from the other cultivars by having the highest values for total phenolic content (PheCo) and chlorophyll contents during the pod formation period (ToChPoSt, ChAPoPe, ChBPoPe). It was determined that Arıoğlu 2003 and Flower 22 cultivars exhibited a similar clustering in terms of flavonoid content (FlaCo) and antioxidant activity (AnOxDaAc). Furthermore, heat map analysis revealed that the Osmaniye 2005 and Sultan cultivars are the most suitable parental candidates for breeding programs focused on high yield, while the Brantley cultivar is the most suitable for studies focused on high oil quality and oxidative stability (**Figure 4**).

### 3.7 Principal component analysis

**Figure 5:**
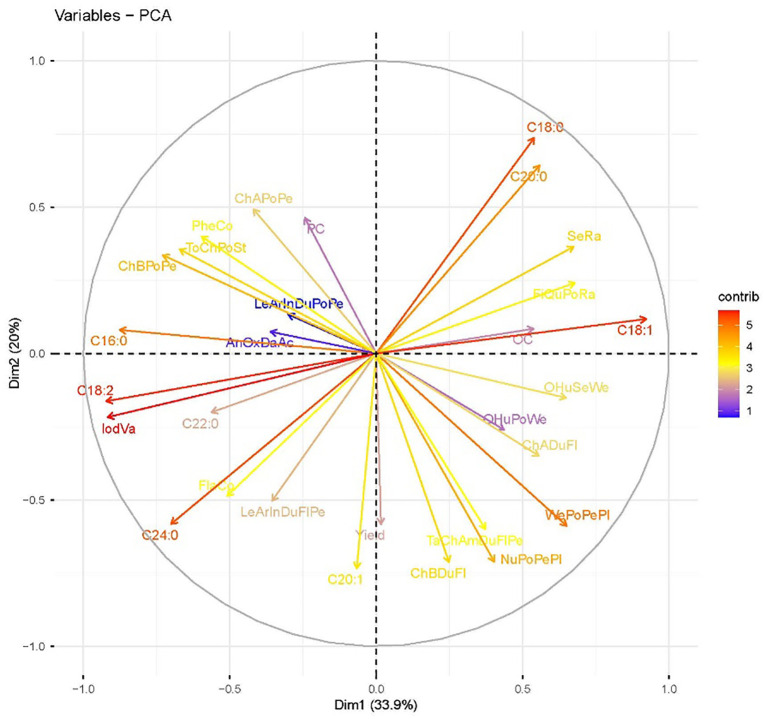
Principal component analysis (PCA) biplot showing the contribution of variables to the total variance and the differentiation of peanut traits based on the first two dimensions.

The results of the Principal Component Analysis (PCA), conducted to evaluate the relationships among all agronomic, quality, and physiological parameters examined using a holistic approach and to determine the contributions of the variables to the total variation, are presented in **Figure 5**. The analysis revealed that the first two principal components (Dim1 and Dim2) accounted for 53.9% of the total variation (33.9% and 20.0%, respectively). Examination of the PCA biplot graph clearly illustrates the vector lengths and directions of the variables, the correlations among the parameters, and their effectiveness along the Dim1-Dim2 axes. While oleic acid (C18:1) had the highest positive effect on the first principal component (Dim1) (dark red vectors), linoleic acid (C18:2), the iodine value (IV), and palmitic acid (C16:0) were found to lie in the negative region of Dim1. This finding indicates that the Dim1 axis is primarily associated with “oil quality and oxidative stability.” It confirmed the negative relationship between oleic acid and linoleic acid. The second principal component (Dim2), on the other hand, is predominantly concentrated around “yield components and physiological parameters.” It was found that fruit yield (Yield), number of fruits per plant (NuPoPePl), fruit weight per plant (WePoPePl), and chlorophyll contents during the flowering period (TaChAmDuFlPe, ChBDuFl) clustered in the negative region of Dim2. However, chlorophyll contents during the fruit formation period (ChAPoPe, ChBPoPe, ToChPoSt) and total phenolic content (PheCo) were found to be located in the positive region of Dim2. When the angles between the vectors were evaluated, a close-angled (positive correlation) relationship was found between pod yield (Yield), the number of pod per plant (NuPoPePl), and eicosenoic acid (C20:1). Additionally, it was observed that the parameters oil content (OC) shelling ratio (SeRa), and first quality pod ratio (FiQuPoRa) exhibited a similar trend to oleic acid (C18:1) in the positive region of Dim1. However, PCA analysis revealed that yield and quality parameters in peanuts are grouped under different components, and these groupings should be considered in breeding strategies (**Figure 5**).

### 3.8 Three-dimensional interaction analysis of yield, physiological, and bioactive parameters

**Figure 6:**
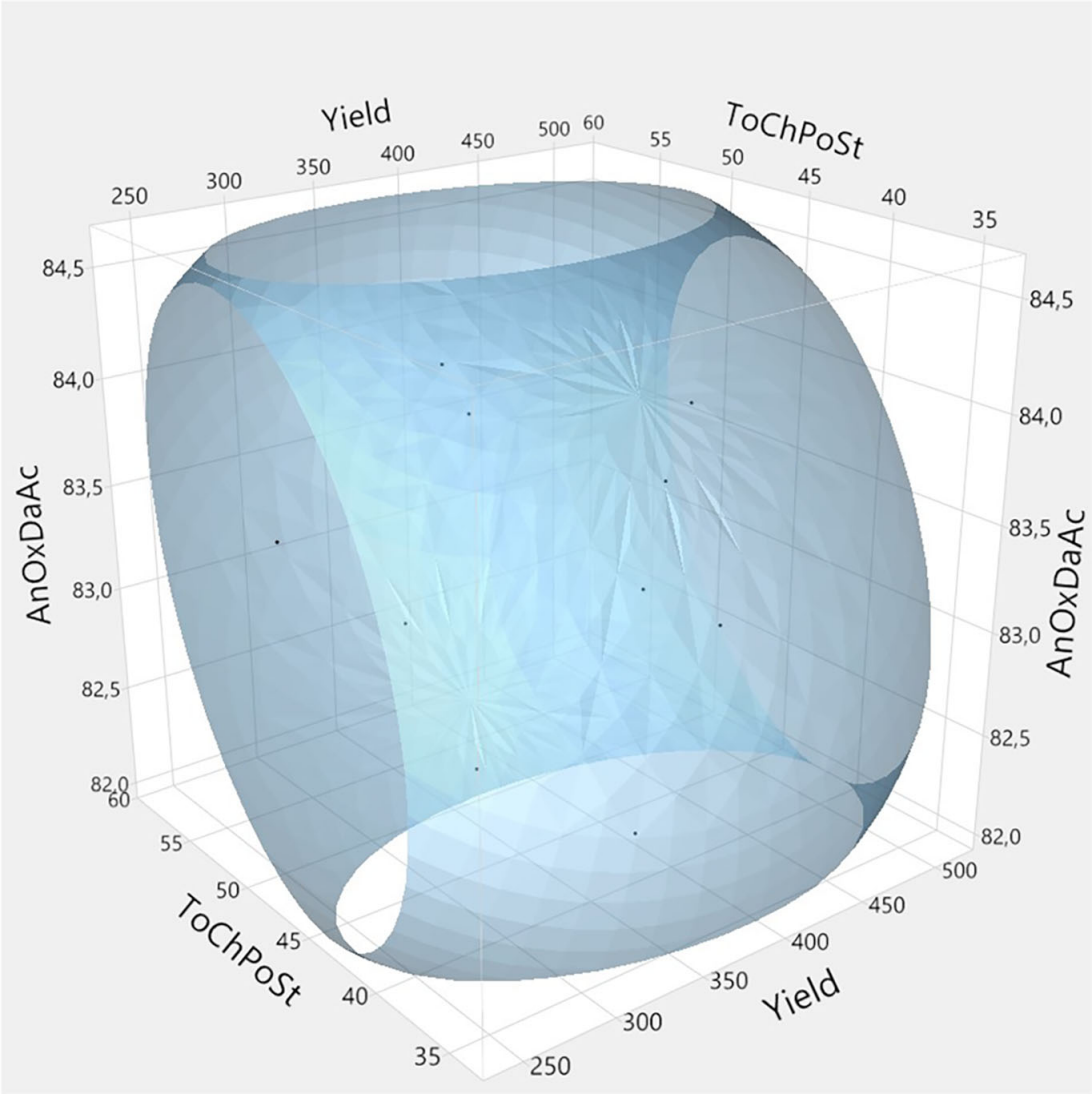
Three-dimensional response surface plot showing the interaction and spatial distribution of yield, chlorophyll content, and antioxidant activity across the evaluated cultivars.

A three-dimensional (3D) interaction analysis graph, created to determine the relationships between pod yield (Yield), total chlorophyll content during the pod formation period (ToChPoSt), and total antioxidant activity (AnOxDaAc), as well as the effects of these parameters on each other, is presented in **Figure 6**. The 3D modeling results revealed non-linear relationships between pod yield and physiological and biochemical characteristics, indicating that these characteristics interact within the same framework. Upon examining the surface trend in the graph, it was observed that in regions where pod yield rose to levels of 4500-5000 kg ha^−1^, the total chlorophyll content during the pod formation period concentrated in the range of 45–55, and this increase elevated the total antioxidant activity to levels of 83.5–84.5%. In particular, the increase in antioxidant capacity alongside higher yields demonstrates that high-yielding cultivars possess strong bioactive potential. The peaks on the surface represent the “ideal zone” where the combination of optimal yield and chlorophyll content maximizes antioxidant activity. This three-dimensional interaction model shows that yield increases in peanuts are determined not only by agronomic factors but also by photosynthetic capacity and high bioactive content (**Figure 6**).

## 4 Discussion

### 4.1 Genotypic and environmental effects on pod yield and yield components

The wide variation in pod yield values observed among cultivars in this study indicates that the peanut materials examined possess different genetic potentials and environmental adaptation capabilities. The high yield performance demonstrated by the Osmaniye 2005 and Sultan cultivars stems from their ability to optimize key yield components such as the number of pod per plant and pod weight (Aytekin and Çalışkan, 2016; Şahin et al., 2022). Therefore, the positive correlation between yield and the number of pod per plant confirms that pod-setting capacity is the primary determinant in increasing yield per unit area in peanuts (Aşık et al., 2018). This indicates that high-yielding cultivars demonstrate more effective pollination and fertilization success during the flowering period (Otieno et al., 2020; Garibaldi et al., 2021). The higher yield in 2023 compared to 2024 can be explained by climatic differences during the trial years, particularly the effects of temperature and humidity regimes during the flowering and pod-filling stages on photosynthetic efficiency (Aşık and Arıoğlu, 2020; Keten et al., 2023; Yılmaz, 2023). It is known that excessive temperatures during the pod filling stage limit assimilate accumulation, thereby exerting pressure on thousand-seed weight (Puppala et al., 2023). The significant C × Y interaction (*p* < 0.01) observed in this study reveals that cultivars responded differently to inter-annual climatic variation. Osmaniye 2005 and Sultan cultivars maintained consistently high pod yields across both seasons, Brantley exhibited greater yield instability, suggesting a narrower environmental adaptation range (Beycioglu and Özcan, 2025; Yılmaz 2023).

### 4.2 Changes in oil, protein, and fatty acid composition

In peanuts, oil content and fatty acid composition are a complex process controlled by both genetic structure and environmental factors. The fact that the Brantley cultivar possesses the highest oil content and oleic acid ratio demonstrates this cultivar’s genetic superiority in oil synthesis metabolism (Nawade et al., 2016; Yılmaz et al., 2023). The negative correlation observed between oleic acid (C18:1) and linoleic acid (C18:2) stems from differences in oleate desaturase (FAD2) enzyme activity among cultivars (Mei et al., 2020). The reduction in FAD2 enzyme activity inhibits the conversion of oleic acid to linoleic acid, leading to high accumulation of oleic acid in the seed (Tonnis et al., 2020). In cultivars with high oleic acid content, the reduction in linoleic acid synthesis is a key quality parameter that enhances the oil’s oxidative stability and extends its shelf life (Wang et al., 2018). This biochemical difference benefits the food industry by increasing peanut oil’s resistance to rancidity (Otyama et al., 2021). The lowest iodine value obtained in the Brantley cultivar is directly related to the decrease in the degree of unsaturation. This situation enhances the industrial value of peanut cultivars with high oleic acid content (Yılmaz et al., 2023). Although oil content showed no statistically significant year effect (F = 4.16, p > 0.05), the significant C × Y interaction observed for fatty acid composition indicates that the expression of FAD2-mediated oleic/linoleic acid balance is sensitive to annual environmental variation, and that the genetic superiority of high-oleic cultivars such as Brantley may be more pronounced under specific climatic conditions (Skovbjerg et al., 2020).

### 4.3 Correlation of physiological parameters with yield and adaptation

Leaf Area Index (LAI) and chlorophyll content are the key physiological parameters that determine a plant’s photosynthetic potential. The fact that the Halisbey and Osmaniye 2005 cultivars have high LAI values indicates that these cultivars maximize assimilate production by creating a larger photosynthetic surface area (Baslam et al., 2020). High LAI values promote biomass production by enabling the plant canopy to absorb light more effectively (Digrado et al., 2020). The positive correlation between plant chlorophyll content during the pod formation period and yield parameters compared to the flowering period highlights the decisive role of photosynthetic continuity during the pod filling stage in peanut yield (Cooney et al., 2021). Despite the high LAI values obtained during the 2024 growing season, the fact that yield was at lower levels compared to 2023 can be interpreted as excessive vegetative growth potentially limiting the transport of assimilates to reproductive organs (Simkin et al., 2020). The higher chlorophyll content recorded during the pod formation stage compared to the flowering stage across cultivars suggests that sustained photosynthetic activity during the reproductive phase is a critical determinant of pod filling efficiency and final yield in peanuts (Shoman, 2024). This finding is consistent with the view that chlorophyll retention during late growth stages reflects a "stay-green" characteristic associated with higher yield potential in legume crops (Silva et al. 2024). Furthermore, the significant C × Y interaction observed for LAI and chlorophyll parameters indicates that the physiological response of cultivars to inter-annual environmental variation is genotype-dependent. Cultivars such as Halisbey and Osmaniye 2005, which maintained high chlorophyll content during the pod formation stage across both years, demonstrated greater physiological stability and consequently more consistent yield performance (Sun et al. 2025).

### 4.4 The importance of bioactive compounds and antioxidant capacity

The phenolic and flavonoid compounds in peanut seeds play a role in the plant’s defense mechanism against biotic and abiotic stresses. Additionally, they contribute to the antioxidant value of the plant in human nutrition. The fact that the Wilson cultivar stands out in terms of total phenolic content indicates that it possesses a high capacity for secondary metabolite synthesis (Massarioli et al., 2022). The high concentration of phenolic compounds in the seed creates a natural protective barrier against lipid peroxidation during seed storage (Zou et al., 2024). The positive correlations between total antioxidant activity and chlorophyll content can be explained by the protective role of the antioxidant system in safeguarding photosynthetic pigments (Juliano et al., 2019). As seen in the 3D surface response modeling, maintaining antioxidant capacity at a certain level alongside yield increases indicates that high-yielding cultivars possess a metabolically more balanced defense system and establish a positive yield-quality balance.

### 4.5 Evaluation of multivariate analyses in terms of breeding strategies

In the PCA analysis, the fact that oleic acid, linoleic acid, and iodine values are positioned in opposite directions along the Dim1 axis indicates that these parameters are important variables in peanut quality breeding (Tiwari et al., 2021). An examination of the heat map reveals that the Brantley cultivar has a higher oleic acid content than the other cultivars, highlighting its genetic distinctiveness within the population. Furthermore, the yield-focused clustering of the Osmaniye 2005 and Sultan cultivars suggests that these cultivars have a similar genetic structure or adaptive capabilities (Dama et al., 2026). These multivariate approaches will provide a database for parent selection to develop both high-yielding and high-quality lines in future breeding programs (Yol et al., 2018; Janila et al., 2016).

## 5 Conclusions

This study has revealed a wide genetic diversity among peanut cultivars in terms of yield, quality, physiological, and bioactive parameters under the ecological conditions of Denizli. According to the results obtained, a positive correlation was found between pod yield and the number of pods per plant as well as pod weight. Additionally, it was found that high yield potential is directly related to photosynthetic capacity (LAI and chlorophyll content) during the flowering and pod formation periods. When the cultivars were examined biochemically, the Brantley cultivar stood out with its high oleic acid content and low iodine value. This indicates oil quality and oxidative stability. Furthermore, a negative correlation was observed between the oleic and linoleic acid ratios among the cultivars. However, the high phenolic compound content of the Wilson cultivar and the antioxidant capacity of the Flower 22 cultivar indicate that these cultivars are important production materials for the functional food industry. Multivariate analyses (PCA and Heat Map) indicate that the cultivars are clustered based on yield and quality. Osmaniye 2005 and Sultan cultivars were identified as the most ideal parental candidates for yield-focused breeding programs, while the Brantley cultivar was identified as the most ideal for quality-focused breeding programs. When examining the 3D surface response model, the positive correlations between yield, chlorophyll content, and antioxidant activity will provide an important perspective for future selection criteria. In conclusion, this study will form the basis for the genetic materials and selection criteria to be used in the development of peanut cultivars with both high yield and high oil quality in the region.

## Data Availability

The original contributions presented in the study are included in the article/supplementary material. Further inquiries can be directed to the corresponding author.
